# Association Between Lactate and ICU‐Acquired Infection in Critically Ill Patients With Sepsis: A Retrospective Study Using the MIMIC‐IV Database

**DOI:** 10.1111/jcmm.71090

**Published:** 2026-03-23

**Authors:** Yue Zhao, Lu Yao, Jiaji Fu, Mei He, Peichi Shi, Jiqian Xu, You Shang

**Affiliations:** ^1^ Department of Critical Care Medicine, The First Affiliated Hospital of USTC, Division of Life Science and Medicine University of Science and Technology of China Hefei Anhui China; ^2^ Department of Critical Care Medicine, Union Hospital Tongji Medical College, Huazhong University of Science and Technology Wuhan Hubei China; ^3^ Hubei Jiang Xia Laboratory Wuhan Hubei China

**Keywords:** ICU‐acquired infection, lactate, MIMIC database, sepsis

## Abstract

Sepsis is a leading cause of Intensive Care Unit (ICU) mortality, often complicated by secondary infections due to sepsis‐induced immunosuppression. The ICU‐acquired infection (IAI) is of particular concern in severe cases, as it heightens the risk of progression to chronic critical illness (CCI). Given the scarcity of established biomarkers for IAI, this study aimed to evaluate the potential association of early blood lactate levels, a recognised prognostic marker in sepsis, with the occurrence of this complication. We conducted a retrospective analysis of data from the Medical Information Mart for Intensive Care (MIMIC)‐IV database (v3.0), enrolling 17,209 patients. The cohort had a median age of 67 years (IQR, 56–77) and a median admission lactate level of 2.6 mmol/L (IQR, 1.6–4.8). Our analysis revealed a linear dose–response relationship between admission lactate levels and IAI risk. After multivariable adjustment, hyperlactatemia (> 6 mmol/L, i.e., the group with the highest lactate levels, Q5) remained independently associated with increased odds of IAI (OR = 1.36; 95% CI, 1.08–1.70). Notably, while elevated lactate predicted higher 28‐day ICU mortality in patients without IAI, this relationship was not maintained in the IAI cohort following multivariable adjustment.

**Abbreviations**
APSIII: acute physiology score III
APTT: activated partial thromboplastin time
BE: base excess
BMI: body mass index
BUN: blood urea nitrogen
CCI: chronic critical illness
CDC: Centers for Disease Control and Prevention
CI: confidence interval
CITI program: Collaborative Institutional Training Initiative program
DC: dendritic cell
GCS: Glasgow Coma Scale
HR: hazard ratio
HR: heart rate
IAI: ICU‐acquired infection
ICU: intensive care unit
IMV: invasive mechanical ventilation
INR: international normalised ratio
IQRs: interquartile ranges
Lac: lactate
LODS: logistic organ dysfunction score
LOS: length of stay
MBP: mean arterial pressure
mDC: myeloid dendritic cell
mHLA‐DR: monocytic human leukocyte antigen–antigen D related
MIMIC‐IV: Medical Information Mart for Critical Care IV
MODS: multiple organ dysfunction syndrome
ND6: nicotinamide adenine dinucleotide dehydrogenase subunit 6
NFAT: nuclear factor of activated T cell
NIH: National Institutes of Health
NKc: Natural killer cell
NNIS: National Hospital Infection Surveillance
OR: odds ratio
PCO_2_
: partial pressure of carbon dioxide
PO_2_
: partial pressure of oxygen
PT: prothrombin time
RCS: restricted cubic spline
RR: respiration rate
RRT: renal replacement therapy
SAPSII: simplified acute physiology score II
SIRS: systemic inflammatory response syndrome score
SOFA: sequential organ failure assessment
SpO2: percutaneous arterial oxygen saturation
SQL: Structured Query Language
SSC: Surviving Sepsis Campaign
TAMs: tumour‐associated macrophages
TME: tumour microenvironment
Treg: regulatory T cell
TREM‐1: triggering receptor expressed on myeloid cells‐1
WBC: white blood cell

## 1 Introduction

Sepsis is clinically termed a life‐threatening organ dysfunction characterised by a dysregulated host response to an infection [1]. Global epidemiological data from 2017 indicate that sepsis affected approximately 48.9 million individuals worldwide, with a mortality rate of 22.5%, accounting for 20% of all global deaths [2, 3]. The pathophysiology of sepsis is highly complex. In the early stages of the disease, the pro‐inflammatory and anti‐inflammatory phases of the host immune response often occur simultaneously. The accumulation of pro‐inflammatory cytokines is associated with early mortality, while late (chronic) sepsis‐related deaths are frequently linked to marked immunosuppression and immune dysfunction [4]. In fact, more than 70% of sepsis‐related deaths occur after the first three days of onset [5]. Secondary infection is a common complication of immunosuppression. Among sepsis patients, ICU‐acquired infection (IAI) is more prevalent in those with higher disease severity [6, 7]. In recent years, in‐hospital mortality due to sepsis has decreased substantially [8]. However, this reduction has not translated into improved long‐term outcomes. On the contrary, a growing number of sepsis survivors develop chronic critical illness (CCI), which often leads to poor long‐term prognosis and increased healthcare costs [8, 9]. Research has shown that sepsis patients who experience secondary infections are more likely to develop CCI [10], further underscoring the importance of recognising secondary infection as a key outcome indicator in sepsis. Therefore, identifying risk factors that predict the occurrence of secondary infections in sepsis patients is crucial for reducing treatment challenges in critically ill patients and for assessing long‐term prognosis.

Low monocyte HLA‐DR (mHLA‐DR) expression is a widely recognised surrogate marker of sepsis‐induced immunosuppression [11]. In patients with sepsis or septic shock, persistently low mHLA‐DR levels are independently associated with the development of secondary infections—a finding consistent with observations in paediatric septic shock patients [12, 13, 14, 15]. Triggering receptor expressed on myeloid cells‐1 (TREM‐1) is an innate immune receptor involved in cellular activation, and its soluble form sTREM‐1 is considered a strong predictor of sepsis mortality. Combining sTREM‐1 with mHLA‐DR may improve the identification of immunosuppressed patients at risk of nosocomial infections [16]. Although mHLA‐DR is well‐studied and supported by standardised clinical assays, its clinical utility is limited by the requirement for serial measurements over several days to confirm persistent immunosuppression, which reduces its practicality and timeliness. Similarly, other markers—such as persistently low circulating myeloid dendritic cell (mDC) counts, delayed overexpression of S100A9 messenger RNA in whole blood, and elevated expression of PD‐1, PD‐L1, and PD‐L2 on monocytes and CD4^+^ T lymphocytes—have been linked to IAI but also require repeated dynamic monitoring for effective prediction [17, 18, 19, 20]. A recent study found that high circulating levels of nicotinamide adenine dinucleotide dehydrogenase subunit 6 (ND6) at admission were independently and strongly associated with secondary infections, suggesting that ND6 may serve as a promising biomarker for predicting post‐sepsis complications [21]. Further research is needed to explore its predictive value and to develop standardised detection and evaluation methods.

Lactate, a classic byproduct of glucose metabolism, plays a pivotal role in cellular homeostasis, biological function, and immune modulation. For patients with suspected or confirmed sepsis, lactate measurement upon admission has become a standard practice and can be performed rapidly at the bedside in many ICUs, providing results within minutes. Elevated lactate levels are independently associated with organ dysfunction in sepsis [22]. Furthermore, the clinical utility of lactate measurement extends to its ability to predict mortality and identify patients at elevated risk of death. Studies have demonstrated that both acute‐phase and in‐hospital mortality increase linearly with rising lactate levels [23]. A prehospital lactate level > 3 mmol/L enhances early identification of sepsis patients at heightened mortality risk in the emergency department [24]. Given the close association of both elevated lactate levels and secondary infections with disease severity and poor outcomes in sepsis patients, this study hypothesises that higher lactate levels may be correlated with the occurrence of secondary infections in this population. Using data from the Medical Information Mart for Intensive Care IV (MIMIC‐IV, version 3.0) database, we aim to investigate the relationship between lactate levels and IAI in sepsis patients, with the objective of identifying potential novel risk factors for secondary infections.

## 2 Materials and Methods

### 2.1 Source of Data

MIMIC‐IV is a longitudinal, single‐center database comprising 247,366 individuals (including 196,527 adults) admitted to the Beth Israel Deaconess Medical Center between 2008 and 2019. Access to these data was granted after completing the required training course provided by the National Institutes of Health (NIH) and successfully passing the Collaborative Institutional Training Initiative (CITI) program. Personal information was de‐identified within the database to protect patient privacy, which facilitated the waiver of informed consent requirements.

### 2.2 Study Design and Population

**FIGURE 1:**
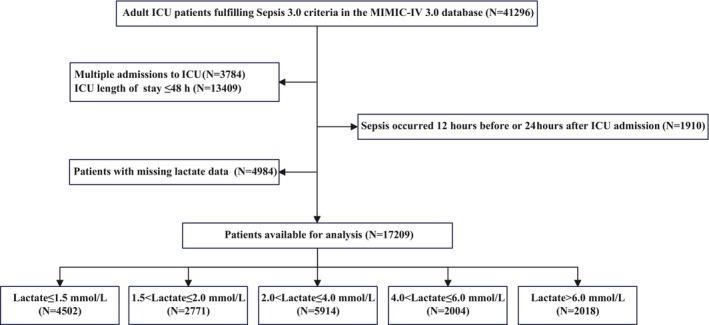
Flow of included patients through the trial. ICU, intensive care unit.

We analysed 41,296 patients diagnosed with sepsis and aged over 18 years from the database. Patients were excluded based on the following criteria: (1) multiple ICU admissions (*n* = 3784); (2) ICU stays ≤ 48 h (*n* = 13,409); (3) sepsis onset occurring 12 h before or 24 h after ICU admission (*n* = 1910) [6]; (4) lack of lactate measurements within the first 24 h of ICU admission (*n* = 4984) (Figure 1). Ultimately, 17,209 patients were included in the study and stratified into five groups according to hyperlactatemia severity defined in previous studies [25, 26, 27, 28, 29]: Q1: ≤ 1.5 mmol/L (*n* = 4502), Q2: > 1.5 and ≤ 2.0 mmol/L (*n* = 2771), Q3: > 2.0 and ≤ 4.0 mmol/L (*n* = 5914), Q4: > 4.0 and ≤ 6.0 mmol/L (*n* = 2004), Q5: > 6.0 mmol/L (*n* = 2018).

Sepsis diagnosis followed the Sepsis‐3 criteria: documented infection (with signs of infection and treated with antibiotics for more than 48 h) plus an increase in Sequential Organ Failure Assessment (SOFA) score of 2 points or more [1]. IAI were defined according to the National Hospital Infection Surveillance (NNIS) System of the U.S. Centers for Disease Control and Prevention (CDC) [6, 30]. For accurate operationalization in the MIMIC‐IV database, a case was confirmed only when the following two conditions were jointly met after 48 h of ICU admission: (1) new microbiological evidence, either a new infection site or a novel pathogen at a prior site, and (2) subsequent initiation of a targeted antimicrobial therapy adjustment in response to the above evidence. Upon meeting the pre‐defined inclusion criteria, patients were categorised according to their documented IAI diagnosis status into the IAI group and the non‐IAI group for analysis.

### 2.3 Data Extraction

Data were extracted using Structured Query Language (SQL) via Navicat Premium (Version 16). Only sepsis patients admitted to the ICU for the first time were included to avoid duplicate entries. Baseline characteristics (age, gender, ethnicity, Body mass index [BMI]), comorbidities (cardiovascular, neurological, pulmonary, hepatic, renal, diabetes, malignancy), and ICU severity scores (Charlson Comorbidity Index, Logistic Organ Dysfunction System [LODS], Oxford Acute Severity of Illness Score [OASIS], Acute Physiology Score III [APSIII], Simplified Acute Physiology Score II [SAPSII], Systemic Inflammatory Response Syndrome [SIRS], Glasgow Coma Scale [GCS], Sequential Organ Failure Assessment [SOFA]) were recorded. Vital signs (temperature, heart rate, mean arterial pressure, respiratory rate, oxygen saturation), laboratory parameters (lactate levels, microbial cultures), and treatments (mechanical ventilation, vasopressors, renal replacement therapy, antibiotics) during the first 24 h in the ICU were analysed (Table S1). Comorbidities were diagnosed using ICD‐9/ICD‐10 codes in the MIMIC‐IV database (v3.0).

### 2.4 Study Endpoint

The primary study endpoint was the incidence of IAI. Secondary objectives included hospital and ICU length of stay, in‐hospital mortality, ICU mortality, and 28‐day mortality. Additionally, we explored the association between lactate levels and 28‐day mortality in sepsis patients with and without IAI.

### 2.5 Statistical Analysis

Variables with a missing data proportion exceeding 30% were excluded from the analysis. The missingness for all retained variables is summarised in Table S2. Under the assumption that the data were missing at random, multiple imputation was performed to handle the remaining missing data using the Random Forest algorithm, generating 20 complete datasets. Following imputation, generalised linear models were fitted to each dataset, and the results were pooled to derive overall estimates. The model with the smallest Akaike Information Criterion (AIC) value was selected for final analysis to ensure optimal model fit.

Continuous variables are presented as medians and interquartile ranges (IQRs) and were compared using the Kruskal–Wallis test. Categorical variables were expressed as numbers and percentages. Pearson's *χ* ^2^ test or Fisher's exact test was used for categorical data, as appropriate. The association between lactate, IAI, and 28‐day mortality was evaluated with continuous‐scale restricted cubic spline (RCS) curves. Primary and secondary outcomes were assessed using logistic regression or Cox regression analysis (with proportional hazards assumption verified using Schoenfeld residuals), adjusting for different factors to build multiple models. Kaplan–Meier survival curves were used to evaluate differences in 28‐day survival rates between groups, and the log‐rank test with Bonferroni correction was applied to account for multiple comparisons.

All *p* values were two‐tailed, and a *p* value < 0.05 was considered statistically significant. Statistical analyses were performed with R4.4.2.

We note that the IAI/non‐IAI grouping served a descriptive role for presenting the exposure variable's distribution, not for conducting formal group comparisons.

## 3 Results

### 3.1 Baseline Characteristics

A total of 17,209 patients were enrolled in this study. The median age was 67 years (IQR, 56–77), with 10,032 (58.30%) female participants. The median lactate level at admission was 2.6 mmol/L (IQR, 1.6–4.8). Patients were stratified into five groups based on blood lactate concentrations, as previously described. The significant differences in severity scores, laboratory results, and treatment interventions among patient groups with varying lactate levels suggest that elevated lactate may be associated with more severe clinical conditions. Baseline characteristics of these groups are summarised in Table 1.

**TABLE 1:** Baseline characteristics of patients admitted with sepsis classified according to lactate.^a^

Variables	Total (0 ≤ Lac ≤ 89.0) (*N* = 17,209)	Q1 (Lac ≤ 1.5) (*n* = 4502)	Q2 (1.5 < Lac ≤ 2.0) (*n* = 2771)	Q3 (2.0 < Lac ≤ 4.0) (*n* = 5914)	Q4 (4.0 < Lac ≤ 6.0) (*n* = 2004)	Q5 (Lac > 6.0) (*n* = 2018)	*p*
Demographics							
Age, year	67 (56–77)	67 (56–78)	67 (57–77)	68 (57–78)	66 (54–77)	65 (53–75)	< 0.001
Gender, *n* (%)							
Female	10,032 (58.3)	2463 (54.7)	1632 (58.9)	3536 (59.8)	1155 (57.6)	1246 (61.7)	< 0.001
Male	7177 (41.7)	2039 (45.3)	1139 (41.1)	2378 (40.2)	849 (42.4)	772 (38.3)	
BMI^b^, kg/m^2^	27.7 (23.88–32.70)	27.88 (23.82–33.33)	27.64 (23.84–32.51)	27.70 (23.88–32.55)	27.42 (23.99–32.25)	27.59 (23.89–32.52)	0.340
Ethnicity, *n* (%)							
White	11,083 (64.4)	3070 (68.2)	1833 (66.1)	3851 (65.1)	1183 (59.0)	1146 (56.8)	< 0.001
African America	1479 (8.6)	380 (8.4)	250 (9.0)	437 (7.4)	188 (9.4)	224 (11.1)	
Hispanic	621 (3.6)	125 (2.8)	86 (3.1)	223 (3.8)	89 (4.4)	98 (4.9)	
Asian	495 (2.9)	101 (2.2)	66 (2.4)	174 (2.9)	74 (3.7)	80 (4.0)	
Other/unknown	3531 (20.5)	826 (18.3)	536 (19.3)	1229 (20.8)	470 (23.5)	470 (23.3)	
Comorbidities, *n* (%)							
Cardiovascular disease	12,918 (75.1)	3371 (74.9)	2117 (76.4)	4523 (76.5)	1462 (73.0)	1445 (71.6)	< 0.001
Neurological disease	3438 (20.0)	964 (21.4)	564 (20.4)	1168 (19.7)	380 (19.0)	362 (17.9)	0.012
Pulmonary disease	5042 (29.3)	1593 (35.4)	875 (31.6)	1643 (27.8)	493 (24.6)	438 (21.7)	< 0.001
Liver disease	3095 (18.0)	494 (11.0)	429 (15.5)	1025 (17.3)	511 (25.5)	636 (31.5)	< 0.001
Renal disease	4578 (26.6)	1357 (30.1)	760 (27.4)	1482 (25.1)	469 (23.4)	510 (25.3)	< 0.001
Diabetes	5838 (33.9)	1522 (33.8)	906 (32.7)	2006 (33.9)	699 (34.9)	705 (34.9)	0.459
Malignancy	2505 (14.6)	699 (15.5)	406 (14.7)	790 (13.4)	275 (13.7)	335 (16.6)	0.001
Scores of severity							
CCI	5 (3–7)	5 (3–8)	5 (3–7)	5 (3–7)	5 (3–7)	5 (3–8)	0.022
LODS^b^	6 (4–8)	5 (4–7)	6 (4–8)	6 (4–8)	7 (5–9)	8 (6–10)	< 0.001
OASIS^b^	36 (31–42)	35 (30–40)	35 (30–40)	36 (31–41)	37 (32–43)	39 (33–46)	< 0.001
APSIII^b^	52 (39–69)	49 (38–61)	49 (37–64)	51 (37–67)	57 (43–74)	68 (52–88)	< 0.001
SAPSII^b^	42 (34–52)	40 (32–48)	41 (33–49)	42 (34–52)	45 (36–55)	50 (41–62)	< 0.001
SIRS^b^	3 (2–4)	3 (2–3)	3 (2–3)	3 (2–4)	3 (3–4)	3 (3–4)	< 0.001
GCS^b^	15 (13–15)	15 (13–15)	15 (13–15)	15 (13–15)	15 (13–15)	15 (13–15)	< 0.001
SOFA^b^	7 (4–9)	6 (4–8)	6 (4–8)	7 (5–9)	8 (6–11)	10 (7–12)	< 0.001
Vital signs^b^							
Temperature, °C	37.4 (37.0–38.0)	37.4 (37.1–38.1)	37.4 (37.0–38.0)	37.4 (37.0–38.0)	37.4 (37.0–38.1)	37.3 (36.9–37.9)	< 0.001
HR, bpm	106 (92–122)	104 (90–118)	105 (91–121)	105 (92–121)	109 (95–125)	114 (99–131)	< 0.001
MBP, mmHg	57 (50–63)	57 (50–64)	57 (51–63)	57 (50–62)	56 (49–62)	55 (47–61)	< 0.001
RR, bpm	28 (24–33)	28 (24–33)	18 (24–33)	28 (24–33)	28 (24–33)	29 (25–34)	< 0.001
SpO_2_, %	92 (89–95)	92 (89–95)	92 (89–95)	93 (90–95)	93 (90–95)	93 (89–95)	< 0.001
Laboratory results^b^							
PH	7.30 (7.23–7.36)	7.34 (7.26–7.39)	7.33 (7.26–7.38)	7.31 (7.25–7.36)	7.26 (7.19–7.32)	7.17 (7.08–7.26)	< 0.001
PO_2_, mmHg	61 (39–91)	66 (43–96)	63 (40–95)	63 (39–94)	55 (37–83)	49 (35–75)	< 0.001
PCO_2_, mmHg	47 (41–56)	47 (39–57)	46 (40–55)	47 (41–55)	48 (42–56)	50 (42–59)	< 0.001
BE, mmol/L	−4 (−8 to 0)	−1 (−4 to 1)	−2 (−5 to 0)	−3 (−6 to −1)	−7 (−10 to −3)	−12 (−16 to −8)	< 0.001
Anion gap, mEq/L	16 (14–20)	15 (13–18)	16 (13–19)	16 (13–19)	18 (15–22)	23 (18–27)	< 0.001
Bicarbonate, mEq/L	21 (17–23)	22 (19–26)	21 (19–24)	21 (18–23)	19 (16–21)	16 (11–19)	< 0.001
Haemoglobin, g/dL	9.3 (7.9–11.0)	9.4 (8.1–10.9)	9.5 (8.2–11.1)	9.4 (8.0–11.1)	9.2 (7.8–11.0)	8.6 (7.2–10.5)	< 0.001
Platelet, K/μL	158 (105–226)	192 (135–264)	166 (114–235)	151 (106–214)	132 (88–191)	118 (66–179)	< 0.001
WBC, K/μL	14.8 (10.6–20.1)	12.6 (9.2–17.1)	14.2 (10.2–19.0)	15.4 (11.3–20.5)	16.7 (12.0–22.4)	17.7 (12.6–24.9)	< 0.001
APTT, s	34.7 (29.2–49.0)	32.2 (28.1–40.7)	33.3 (28.6–45.6)	34.6 (29.4–46.6)	38.0 (30.1–55.5)	45.1 (33.3–72.8)	< 0.001
PT, s	15.4 (13.4–19.4)	14.3 (12.7–16.7)	14.9 (13.1–17.9)	15.6 (13.6–18.7)	17.1 (14.2–22.4)	19.4 (15.4–27.0)	< 0.001
INR, mEq/L	1.4 (1.2–1.8)	1.3 (1.1–1.5)	1.4 (1.2–1.6)	1.4 (1.2–1.7)	1.6 (1.3–2.1)	1.8 (1.4–2.5)	< 0.001
Creatinine, mg/dL	1.3 (0.9–2.2)	1.2 (0.8–2.2)	1.2 (0.9–2.1)	1.2 (0.9–2.0)	1.4 (1.0–2.2)	1.8 (1.2–2.8)	< 0.001
BUN, mg/dL	26 (17–45)	26 (16–46)	26 (17–46)	25 (17–43)	27 (17–43)	29 (20–47)	< 0.001
Sodium, mEq/L	140 (137–143)	140 (137–143)	140 (137–143)	140 (137–143)	140 (137–143)	141 (138–144)	< 0.001
Potassium, mEq/L	4.6 (4.2–5.2)	4.4 (4.0–4.9)	4.5 (4.1–5.0)	4.6 (4.2–5.2)	4.7 (4.3–5.3)	4.9 (4.4–5.6)	< 0.001
Calcium, mEq/L	7.9 (7.4–8.4)	8.0 (7.5–8.5)	8.0 (7.5–8.5)	8.0 (7.4–8.4)	7.8 (7.3–8.4)	7.7 (7.1–8.3)	< 0.001
Chloride, mEq/L	106 (102–110)	105 (101–109)	106 (102–110)	107 (103–111)	107 (103–111)	107 (102–111)	< 0.001
Glucose, mg/dL	157 (125–214)	143 (117–183)	151 (124–195)	156 (124–209)	177 (136–248)	209 (153–307)	< 0.001
Treatment interventions^b^, *n* (%)							
Arterial catheter	9374 (54.5)	1924 (42.7)	1349 (48.7)	3472 (58.7)	1264 (63.1)	1365 (67.6)	< 0.001
Central venous catheter	10,580 (61.5)	2403 (53.4)	1623 (58.6)	3822 (64.6)	1329 (66.3)	1403 (69.5)	< 0.001
Surgical drain	392 (2.3)	74 (1.6)	36 (1.3)	157 (2.7)	57 (2.8)	68 (3.4)	< 0.001
Urinary catheter	1300 (7.6)	286 (6.4)	205 (7.4)	451 (7.6)	170 (8.5)	188 (9.3)	< 0.001
IMV	10,144 (58.9)	2272 (50.5)	1527 (55.1)	3636 (61.5)	1315 (65.6)	1394 (69.1)	< 0.001
RRT	617 (3.6)	98 (2.2)	48 (1.7)	157 (2.7)	74 (3.7)	240 (11.9)	< 0.001
Glucocorticoid	874 (5.1)	211 (4.7)	133 (4.8)	253 (4.3)	111 (5.5)	166 (8.2)	< 0.001
Immunosuppressant	243 (1.4)	77 (1.7)	29 (1.0)	64 (1.1)	41 (2.0)	32 (1.6)	0.003
Antibiotic	15,371 (89.3)	3908 (86.8)	2462 (88.8)	5331 (90.1)	1832 (91.4)	1838 (91.1)	< 0.001

Abbreviations: APSIII, acute physiology score III; APTT, activated partial thromboplastin time; BE, base excess; BMI, body mass index; BUN, blood urea nitrogen; CCI, Charlson comorbidity index; GCS, Glasgow Coma Scale; HR, heart rate; IMV, invasive mechanical ventilation; INR, international normalised ratio; Lac, lactate; LODS, logistic organ dysfunction score; MBP, mean arterial pressure; PCO_2_, partial pressure of carbon dioxide; PO_2_, partial pressure of oxygen; PT, prothrombin time; RR, respiration rate; RRT, renal replacement therapy; SAPSII, simplified acute physiology score II; SIRS, systemic inflammatory response syndrome score; SOFA, sequential organ failure assessment; SpO_2_, percutaneous arterial oxygen saturation; WBC, white blood cell.

^a^ The unit of lactate is mmol/L.

^b^ On the first day of intensive care unit admission.

### 3.2 Clinical Outcomes

Table 2 compares the clinical outcomes of sepsis patients stratified by blood lactate level. The incidence of IAI showed significant differences among the groups (*p* < 0.001). Specifically, the Q4 and Q5 groups exhibited rates of 9.9% and 13.4%, respectively, which were significantly higher than those in the other three groups, indicating that elevated lactate levels are associated with an increased risk of IAI. Significant differences were also observed in both ICU length of stay and total hospital stay among sepsis patients across the groups (*p* < 0.001). The Q5 group had median ICU and total hospital stays of 5.9 and 13.1 days, respectively, which were significantly longer than those in the other four groups. This suggests that elevated lactate levels may be related to greater clinical complexity and prolonged recovery in sepsis patients. Moreover, marked intergroup differences were noted in mortality outcomes (all *p* < 0.001). In‐hospital mortality increased from 15.9% in Q1 to 36.0% in Q5, ICU mortality rose from 10.6% in Q1 to 29.9% in Q5, and 28‐day mortality increased from 18.6% in Q1 to 37.1% in Q5. These results demonstrate a strong association between elevated lactate levels and short‐term mortality in sepsis patients.

**TABLE 2:** Clinical outcomes of patients admitted with sepsis classified according to lactate.^a^

Variables	Total (0 ≤ Lac ≤ 89.0) (*N* = 17,209)	Q1 (Lac ≤ 1.5) (*n* = 4502)	Q2 (1.5 < Lac ≤ 2.0) (*n* = 2771)	Q3 (2.0 < Lac ≤ 4.0) (*n* = 5914)	Q4 (4.0 < Lac ≤ 6.0) (*n* = 2004)	Q5 (Lac > 6.0) (*n* = 2018)	*p*
IAI, *n* (%)	1482 (8.6)	369 (8.2)	207 (7.5)	436 (7.4)	199 (9.9)	271 (13.4)	< 0.001
LOS ICU, day	4.7 (3.0–8.8)	4.8 (3.0–8.7)	4.4 (2.9–8.1)	4.3 (2.9–8.0)	4.9 (3.2–9.2)	5.9 (3.5–10.9)	< 0.001
LOS hospital, day	11.3 (7.0–19.4)	11.2 (7.2–18.8)	10.9 (6.8–18.7)	10.8 (6.9–18.1)	12.0 (7.4–21.4)	13.1 (7.1–23.0)	< 0.001
In‐hospital mortality, *n* (%)	3621 (21.0)	718 (15.9)	540 (19.5)	1163 (19.7)	473 (23.6)	727 (36.0)	< 0.001
ICU mortality, *n* (%)	2614 (15.2)	477 (10.6)	353 (12.7)	835 (14.1)	346 (17.3)	603 (29.9)	< 0.001
28‐day mortality, *n* (%)	4037 (23.5)	839 (18.6)	605 (21.8)	1333 (22.5)	512 (25.5)	748 (37.1)	< 0.001

Abbreviations: IAI, ICU‐acquired infection; ICU, intensive care unit; Lac, lactate; LOS, length of stay.

^a^ The unit of lactate is mmol/L.

### 3.3 Association Between Lactate and IAI

The incidence of IAI significantly increased with higher lactate levels (Q4–Q5 vs. Q1–Q3: 9.9% and 13.4% vs. 7.4%–8.2%; *p* < 0.001; Table 2). The median time from admission to first infection was 5.3 days (IQR, 3.3–9.2; Table S3). Respiratory tract infections predominated (58.8%, *n* = 871), followed by bloodstream (14.4%, *n* = 209) and urinary tract infections (10.4%, *n* = 154). Gram‐negative bacteria (53.9%, *n* = 799) and Gram‐positive bacteria (48.0%, *n* = 711) were the most common pathogens, with fungi accounting for 6.5% (*n* = 97; Tables S3 and S4). Table S5 provides a detailed overview of all IAI developed by patients admitted to the ICU for sepsis.

Multivariable logistic regression analyses demonstrated that the group with the highest lactate level (Q5) was independently associated with IAI across three models (Table 3):

– Model 1 (demographics and physiology): OR = 1.66 (95% confidence interval [CI], 1.40–1.96).
– Model 2 (Model 1 + comorbidities, severity scores and laboratory results): OR = 1.46 (95% CI, 1.17–1.82).
– Model 3 (Model 2 + interventions): OR = 1.36 (95% CI, 1.08–1.70).

**TABLE 3:** Association between different lactate categories and incidence of first IAI in patients admitted with sepsis.

Categories	Unadjusted model		Model 1^a^		Model 2^b^		Model 3^c^	
	OR (95% CI)	*p*	OR (95% CI)	*p*	OR (95% CI)	*p*	OR (95% CI)	*p*
Q1	Reference		Reference		Reference		Reference	
Q2	0.90 (0.76–1.08)	0.266	0.92 (0.76–1.09)	0.304	0.93 (0.77–1.11)	0.407	0.92 (0.77–1.11)	0.379
Q3	0.89 (0.77–1.03)	0.119	0.89 (0.77–1.02)	0.102	0.88 (0.76–1.03)	0.103	0.85 (0.73–0.99)	0.037
Q4	1.23 (1.03–1.48)	0.022	1.20 (1.00–1.43)	0.054	1.14 (0.94–1.39)	0.188	1.09 (0.89–1.34)	0.391
Q5	1.74 (1.47–2.05)	< 0.001	1.66 (1.40–1.96)	< 0.001	1.46 (1.17–1.82)	0.001	1.36 (1.08–1.70)	0.009

Abbreviations: CI, confidence interval; IAI, ICU‐acquired infection; ICU, intensive care unit; OR, odds ratio.

^a^ Adjusted for age, BMI, gender and ethnicity.

^b^ Adjusted for comorbidities (neurological disease, liver disease and malignancy), scores of severity (CCI, LODS, OASIS, APSIII, SAPSII, SIRS, GCS and SOFA), vital signs (temperature and HR), and laboratory results (PH, PCO_2_, BE, bicarbonate, anion gap, haemoglobin, APTT, sodium and chloride) based on model 1.

^c^ Adjusted for treatment interventions (arterial catheter, surgical drain, urinary catheter, IMV, RRT and antibiotic) based on model 2.

Additionally, we evaluated the potential non‐linear relationship between lactate and IAI using the RCS model. The results (Figure S1) showed that the correlation between lactate and IAI was positive and linear (non‐linear *p* = 0.551, Overall *p* < 0.001). The threshold for increased infection risk was 2.27 mmol/L (OR = 1).

### 3.4 Association Between Lactate and Mortality

**FIGURE 2:**
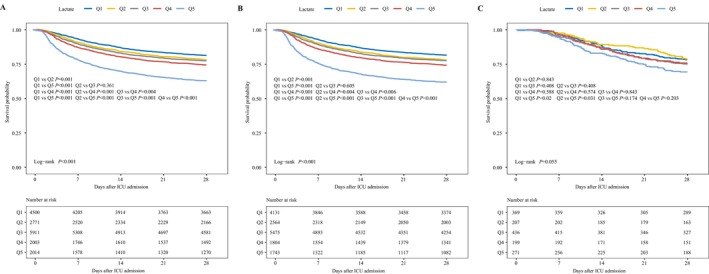
Impact of lactate category on survival outcomes of sepsis patients. (A) Kaplan–Meier survival curves illustrating the effect of different lactate categories on survival outcomes in all patients with sepsis (*N* = 17199). (B) Kaplan–Meier survival curves delineating the influence of different lactate categories on survival outcomes among patients without IAI (*n* = 15179). (C) Kaplan–Meier survival analysis evaluating the influence of different lactate categories on survival outcomes among patients with IAI (*n* = 1482). IAI, ICU‐acquired infection; ICU, intensive care unit. This analysis included a total of 17199 patients with sepsis, of whom 1482 had an IAI.

Figure 2 presents Kaplan–Meier survival curves for different patient cohorts to assess 28‐day mortality. Across all sepsis patient groups, higher lactate levels were associated with lower survival rates. Among patients with non‐IAI, those in the high‐lactate group exhibited a significantly reduced probability of survival. In patients with IAI, the difference in survival was more pronounced in the Q5 group compared to the other groups, particularly when compared to Q1 and Q2 (all *p*‐values < 0.05).

Cox regression models stratified by infection status showed (Table 4):

– Non‐IAI group: Adjusted HRs for 28‐day mortality increased progressively from Q2 (HR = 1.21; 95% CI, 1.08–1.35; Model 1) to Q5 (HR = 2.61; 95% CI, 2.35–2.90; Model 1). This trend persisted after more extensive adjusting for confounders (Model 3: Q2 (HR = 1.13; 95% CI, 1.02–1.27) to Q5 (HR = 1.60; 95% CI, 1.39–1.84).
– IAI group: Initial associations Q5 (HR = 1.51; 95% CI, 1.11–2.05; Model 1) attenuated after full adjustment (Model 3: HR = 0.99; 95% CI, 0.64–1.55), suggesting confounding by ICU interventions or infection‐related factors.

**TABLE 4:** 28‐day mortality in different lactate categories with or without IAI.

		Unadjusted model		Model 1^a^		Model 2^b^		Model 3^c^	
		HR (95% CI)	*p*	HR (95% CI)	*p*	HR (95% CI)	*p*	HR (95% CI)	*p*
28‐day mortality									
Without IAI									
Q1	759/4133 (18.4)	Reference		Reference		Reference		Reference	
Q2	561/2564 (21.9)	1.22 (1.09–1.36)	< 0.001	1.21 (1.08–1.35)	0.001	1.12 (1.00–1.25)	0.041	1.13 (1.02–1.27)	0.025
Q3	1224/5478 (22.3)	1.26 (1.15–1.38)	< 0.001	1.25 (1.14–1.37)	< 0.001	1.14 (1.04–1.26)	0.006	1.17 (1.07–1.29)	0.001
Q4	464/1805 (25.7)	1.49 (1.33–1.67)	< 0.001	1.52 (1.35–1.70)	< 0.001	1.17 (1.04–1.33)	0.012	1.22 (1.08–1.39)	0.002
Q5	665/1747 (38.1)	2.50 (2.25–2.77)	< 0.001	2.61 (2.35–2.90)	< 0.001	1.54 (1.34–1.77)	< 0.001	1.60 (1.39–1.84)	< 0.001
With IAI									
Q1	80/369 (21.7)	Reference		Reference		Reference		Reference	
Q2	44/207 (21.3)	0.96 (0.66–1.39)	0.828	0.97 (0.67–1.41)	0.887	0.95 (0.65–1.37)	0.771	0.96 (0.66–1.39)	0.826
Q3	109/436 (25.0)	1.18 (0.88–1.57)	0.261	1.20 (0.90–1.60)	0.214	1.05 (0.78–1.43)	0.733	1.07 (0.79–1.45)	0.677
Q4	48/199 (24.1)	1.13 (0.79–1.62)	0.497	1.22 (0.85–1.74)	0.278	0.99 (0.66–1.47)	0.954	1.01 (0.67–1.51)	0.969
Q5	83/271 (30.6)	1.51 (1.11–2.05)	0.009	1.51 (1.11–2.05)	0.009	0.96 (0.62–1.50)	0.857	0.99 (0.64–1.55)	0.969

Abbreviations: CI, confidence interval; HR, hazard ratio; IAI, ICU‐acquired infection; ICU, intensive care unit.

^a^ Adjusted for age and gender.

^b^ Adjusted for comorbidities (cardiovascular disease, pulmonary disease, liver disease, renal disease and malignancy), scores of severity (CCI, LODS, OASIS, APSIII, SAPSII and SOFA), vital signs (temperature and SpO_2_), and laboratory results (PH, PO2, BE, bicarbonate, anion gap, haemoglobin, platelet, APTT, PT, INR, creatinine, BUN, potassium, glucose and chloride) based on model 1.

^c^ Adjusted for treatment interventions (arterial catheter, urinary catheter, IMV and RRT) based on model 2.

Elevated lactate levels were independently associated with short‐term mortality in non‐IAI sepsis patients. However, this association was attenuated in IAI sepsis patients, underscoring the multifactorial nature of ICU outcomes. Similar trends were observed for in‐hospital and ICU all‐cause mortality (Table S6).

RCS analysis indicated a nonlinear relationship between lactate and mortality in non‐IAI patients. While the correlation between lactate and 28‐day ICU mortality was positive and linear when patients in the IAI group (non‐linear *p* = 0.711, Overall *p* < 0.001), with mortality risk escalating above 2.62 mmol/L (Figure S2).

## 4 Discussion

This study indicates that, after adjusting for key confounding factors, hyperlactatemia (> 6 mmol/L) is associated with a modest yet statistically significant increase in the risk of IAI in sepsis patients, demonstrating a consistent dose‐dependent upward trend. Notably, the prognostic significance of hyperlactatemia appears to be context‐dependent: while elevated lactate levels show a significant association with increased 28‐day mortality risk in patients without IAI, this association is attenuated in those who develop IAI. Similar patterns were observed for in‐hospital mortality and ICU all‐cause mortality. Collectively, these observational findings suggest a potential dual role of lactate in sepsis—not only as a marker of hypoperfusion but also as a factor possibly linked to an increased risk of specific infectious complications (e.g., IAI). Furthermore, these findings suggest that the interpretation of lactate's prognostic value should be approached with greater caution.

The occurrence of IAI undoubtedly complicates the treatment of sepsis patients and is closely associated with poor clinical outcomes. Based on our findings, a lactate level exceeding 6 mmol/L within the first 24 h of ICU admission is an independent correlate of IAI development and may warrant consideration in clinical assessment. The underlying mechanisms remain unclear. It is well established that sepsis involves dynamic immune phases: initially characterised by predominant pro‐inflammatory activity, followed by a tendency to shift towards predominant anti‐inflammatory activity in the later stages [4]. Current mainstream views suggest that sepsis‐induced immunosuppression is a key factor contributing to secondary infections [26, 31, 32, 33, 34]. Whether lactate influences the occurrence of secondary infections through immunomodulatory mechanisms warrants further investigation.

As early as 2012, blood lactate—a quantitative index reflecting tissue perfusion—was included in the Surviving Sepsis Campaign (SSC) guidelines for managing sepsis and septic shock. Elevated lactate levels in sepsis patients often indicate more severe disease and are associated with poor prognosis. Recently, some researchers have suggested that hyperlactatemia in sepsis may be linked to disruptions in mitochondrial pyruvate metabolism [35]. Emerging evidence suggests that extracellular lactate may regulate immune cell function. For instance, tumour‐derived lactate induces M2‐like polarisation of tumour‐associated macrophages (TAMs) through hypoxia‐inducible factor 1/2α (HIF1/2α)‐mediated mechanisms [36, 37, 38]. Similarly, in inflammatory models, lactate inhibits glycolysis and promotes the transition of LPS‐induced macrophages to a reparative phenotype via PKM2 activation [39]. Recent studies indicate that lactate fuels histone H3K27 acetylation, enabling immunosuppressive gene expression (e.g., Nr4a1) and suppressing macrophage pro‐inflammatory functions [40]. Tumour‐derived lactate can also cause Natural killer cell (NKc) dysfunction through the SIX1/LDHA axis, and decreases NKc activation (downregulation of NKp46, CD25, NKG2D) by suppressing nuclear factor of activated T cell (NFAT) expression due to acidification [41, 42] Malfunction of neutrophil apoptosis is one of the key cellular mechanism of immune suppressive status in sepsis patients. In an in vivo sepsis model, lactate upregulates PD‐L1 on neutrophils via MCT1‐dependent pathways, delaying apoptosis and exacerbating disease severity [43]. Furthermore, lactate inhibits dendritic cell (DC) processes critical for T cell priming, including differentiation, antigen presentation, and activation [42]. Elevated lactate concentrations in the tumour microenvironment (TME) enhance Foxp3 expression, promoting regulatory T cell (Treg) development and immune evasion [44]. In adaptive immunity, lactate disrupts glycolysis by blocking GAPDH and PGDH, depleting intermediates essential for T cell proliferation (e.g., serine). Recent findings also highlight lactate's role in viral infections: viral‐induced lactylation suppresses immune responses by modulating RNA‐binding protein 14 and interferon‐gamma‐inducible protein 16 (IFI16), facilitating immune evasion [45]. In summary, elevated lactate exerts immunosuppressive effects on both innate and adaptive immunity.

The critical role of lactate in sepsis‐induced immunosuppression is well‐established. Our findings provide further support by identifying hyperlactatemia as an independent correlate of IAI. Despite the association being modest in adjusted analysis (OR = 1.36; 95% CI, 1.08–1.70; Model 3), the significant linear dose–response relationship across lactate levels (*p* for trend < 0.001) strengthens the biological plausibility of this link, suggesting it is less likely to be entirely attributable to unmeasured confounders. Nevertheless, the observational nature of our study mandates caution. The modest effect size could still be influenced by residual confounding (e.g., unmeasured nuances of illness severity or source control) or reverse causality, wherein subclinical or early IAI contributes to elevated lactate levels. Therefore, while our data support an associative link, they do not establish causality. Mechanistically, lactate may exacerbate immunosuppression in sepsis through its regulatory effects on immune cell function—a hypothesis that warrants further mechanistic validation. In line with previous research, our study also reaffirms the value of lactate as a biomarker for predicting short‐term mortality in septic patients. However, this predictive power was diminished among sepsis patients with IAI, likely due to their more complex clinical profiles and greater severity of multi‐organ dysfunction. In such cases, the underlying critical illness may become the predominant determinant of mortality, potentially overshadowing the prognostic value of initial lactate levels. Furthermore, the inherent severity of illness in these patients often necessitates more intensive clinical interventions, which can alter lactate dynamics and confound the predictive accuracy of lactate and other established prognostic indicators. These possibilities should be explored in future clinical studies.

This study has several limitations. First, as a single‐center database analysis employing specific exclusion criteria (lack of lactate measurement and ICU stay ≤ 48 h), our study cohort may reflect a patient population with intermediate illness severity from a single geographic region, which could jointly limit the generalisability of our findings. Second, the database contained a substantial number of missing values for variables such as ALT, AST, and albumin. Although these variables were considered important, they were excluded from the analysis to mitigate the impact of missing data. We anticipate that future research, aided by database improvements and advances in artificial intelligence, will address this issue [46]. Third, despite strict inclusion and exclusion criteria, unaccounted factors (e.g., undiagnosed hyperlactatemia prior to ICU admission) might have influenced the results due to database limitations. These potential confounders should be explored in greater depth as the database is improved. Finally, given the retrospective observational nature of this study, establishing a clear causal relationship remains challenging. Therefore, prospective studies with larger sample sizes are warranted to validate these findings.

## 5 Conclusion

Based on our observational data, elevated lactate levels were an independent correlate of IAI in sepsis patients. Furthermore, lactate was associated with an increased risk of short‐term mortality in this population.

## Author Contributions

**Yue Zhao:** data curation (equal), investigation (equal), writing – original draft (lead). **Lu Yao:** data curation (equal), formal analysis (lead), investigation (equal). **Jiaji Fu:** investigation (equal). **Mei He:** investigation (equal). **Peichi Shi:** investigation (equal). **Jiqian Xu:** conceptualization (equal), project administration (equal), supervision (lead), writing – review and editing (equal). **You Shang:** conceptualization (equal), funding acquisition (lead), project administration (equal), writing – review and editing (equal).

## Funding

This study was supported by grants from the National Science and Technology Major Project (2023ZD0506504); the National Natural Science Foundation of China (82372176, 82272217, and 82002026).

## Ethics Statement

The MIMIC‐IV database was approved by the Massachusetts Institute of Technology (Cambridge, MA) and Beth Israel Deaconess Medical Center (Boston, MA). Personal information was de‐identified within the database to protect patient privacy. Written informed consent for participation was not required for this study in accordance with the national legislation and the institutional requirements.

## Conflicts of Interest

The authors declare no conflicts of interest.

## Supporting information


**Figure S1:** Lactate and the estimated probability of first IAI occurrence (*n* = 1482). The restricted cubic spline curve illustrates the association between serum lactate levels (*X*‐axis, mmol/L) and the adjusted odds ratios (OR, with 95% CI) for the first IAI occurrence (*Y*‐axis). Shaded area represents 95% CI. OR = 1, lactate = 2.27 mmol/L. ICU, intensive care unit; CI, confidence interval; IAI, ICU‐acquired infection; OR, odds ratio. This analysis included 1482 patients with sepsis and IAI.

**Figure S2:** Relationship between lactate and 28‐day mortality. The three restricted cubic spline curves display the association between serum lactate levels (*X*‐axis, mmol/L) and the adjusted hazard ratio (HR, with 95% CI) for 28‐day mortality (*Y*‐axis) across the specified patient populations. (A) In all sepsis populations, lactate was 2.27 mmol/L with HR = 1 (*N* = 17199). (B) In sepsis patients without IAI, lactate was 2.25 mmol/L with HR = 1 (*n* = 15179). (C) In sepsis patients with IAI, lactate was 2.62 mmol/L with HR = 1 (*n* = 1482). Shaded areas represent 95% CI. CI, confidence interval; HR, hazard ratio; IAI, ICU‐acquired infection; ICU, intensive care unit. This analysis included a total of 17199 patients with sepsis, of whom 1482 had an IAI.

**Table S1:** Selection strategy for variables with multiple measurements.

**Table S2:** Missing rate for variables extracted from MIMIC‐IV 3.0.

**Table S3:** Infection information of first IAI in patients admitted with sepsis classified according to lactate.^a^

**Table S4:** Detailed isolated microorganisms of first IAI in patients admitted with sepsis classified according to lactate.^a^

**Table S5:** Detailed infection information of all IAI in patients admitted with sepsis classified according to lactate.^a^

**Table S6:** ICU and in‐hospital mortality in different lactate categories with or without IAI.

## Data Availability

The data that support the findings of this study are available from the corresponding author upon reasonable request.

## References

[1] M. Singer , C. S. Deutschman , C. W. Seymour , et al., “The Third International Consensus Definitions for Sepsis and Septic Shock (Sepsis‐3),” JAMA 315, no. 8 (2016): 801–810, 10.1001/jama.2016.0287. 26903338 PMC4968574

[2] K. E. Rudd , S. C. Johnson , K. M. Agesa , et al., “Global, Regional, and National Sepsis Incidence and Mortality, 1990–2017: Analysis for the Global Burden of Disease Study,” Lancet 395, no. 10219 (2020): 200–211, 10.1016/s0140-6736(19)32989-7. 31954465 PMC6970225

[3] C. W. Seymour , J. N. Kennedy , S. Wang , et al., “Derivation, Validation, and Potential Treatment Implications of Novel Clinical Phenotypes for Sepsis,” JAMA 321, no. 20 (2019): 2003–2017, 10.1001/jama.2019.5791. 31104070 PMC6537818

[4] R. S. Hotchkiss , G. Monneret , and D. Payen , “Sepsis‐Induced Immunosuppression: From Cellular Dysfunctions to Immunotherapy,” Nature Reviews. Immunology 13, no. 12 (2013): 862–874, 10.1038/nri3552. PMC4077177 24232462

[5] G. P. Otto , M. Sossdorf , R. A. Claus , et al., “The Late Phase of Sepsis Is Characterized by an Increased Microbiological Burden and Death Rate,” Critical Care 15, no. 4 (2011): 8, 10.1186/cc10332. PMC3387626 21798063

[6] L. A. van Vught , P. M. Klein Klouwenberg , C. Spitoni , et al., “Incidence, Risk Factors, and Attributable Mortality of Secondary Infections in the Intensive Care Unit After Admission for Sepsis,” JAMA 315, no. 14 (2016): 1469–1479, 10.1001/jama.2016.2691. 26975785

[7] G. J. Zhao , D. Li , Q. Zhao , et al., “Incidence, Risk Factors and Impact on Outcomes of Secondary Infection in Patients With Septic Shock: An 8‐Year Retrospective Study,” Scientific Reports 6 (2016): 9, 10.1038/srep38361. 27924831 PMC5141415

[8] K. M. Kaukonen , M. Bailey , S. Suzuki , D. Pilcher , and R. Bellomo , “Mortality Related to Severe Sepsis and Septic Shock Among Critically III Patients in Australia and New Zealand, 2000–2012,” JAMA: The Journal of the American Medical Association 311, no. 13 (2014): 1308–1316, 10.1001/jama.2014.2637. 24638143

[9] S. Yende , S. Austin , A. Rhodes , et al., “Long‐Term Quality of Life Among Survivors of Severe Sepsis: Analyses of Two International Trials,” Critical Care Medicine 44, no. 8 (2016): 1461–1467, 10.1097/ccm.0000000000001658. 26992066 PMC4949079

[10] P. Efron , J. Stortz , H. Horiguchi , et al., “The Immune Status and Outcomes of Septic Patients Who Develop Secondary Infections,” Critical Care Medicine 46, no. 1 (2018): 714, 10.1097/01.ccm.0000529463.06958.48.

[11] L. K. Torres , P. Pickkers , and T. van der Poll , “Sepsis‐Induced Immunosuppression,” Annual Review of Physiology 84 (2022): 157–181, 10.1146/annurev-physiol-061121-040214. 34705481

[12] C. Landelle , A. Lepape , N. Voirin , et al., “Low Monocyte Human Leukocyte Antigen‐DR Is Independently Associated With Nosocomial Infections After Septic Shock,” Intensive Care Medicine 36, no. 11 (2010): 1859–1866, 10.1007/s00134-010-1962-x. 20652682

[13] A. M. Drewry , E. A. Ablordeppey , E. T. Murray , et al., “Comparison of Monocyte Human Leukocyte Antigen‐DR Expression and Stimulated Tumor Necrosis Factor Alpha Production as Outcome Predictors in Severe Sepsis: A Prospective Observational Study,” Critical Care 20 (2016): 10, 10.1186/s13054-016-1505-0. 27760554 PMC5072304

[14] C. de Roquetailla , C. Dupuis , V. Faivre , A. C. Lukaszewicz , C. Brumpt , and D. Payen , “Monitoring of Circulating Monocyte HLA‐DR Expression in a Large Cohort of Intensive Care Patients: Relation With Secondary Infections,” Annals of Intensive Care 12, no. 1 (2022): 9, 10.1186/s13613-022-01010-y. 35526199 PMC9079217

[15] S. Remy , K. Kolev‐Descamps , M. Gossez , et al., “Occurrence of Marked Sepsis‐Induced Immunosuppression in Pediatric Septic Shock: A Pilot Study,” Annals of Intensive Care 8 (2018): 10, 10.1186/s13613-018-0382-x. 29536210 PMC5849582

[16] M. Venet , F. Bidar , M. Derive , et al., “Persistently Elevated Soluble Triggering Receptor Expressed on Myeloid Cells 1 and Decreased Monocyte Human Leucocyte Antigen DR Expression Are Associated With Nosocomial Infections in Septic Shock Patients,” Critical Care Explorations 5, no. 3 (2023): 12, 10.1097/cce.0000000000000869. PMC9970267 36861044

[17] D. Grimaldi , S. Louis , F. Pène , et al., “Profound and Persistent Decrease of Circulating Dendritic Cells Is Associated With ICU‐Acquired Infection in Patients With Septic Shock,” Intensive Care Medicine 37, no. 9 (2011): 1438–1446, 10.1007/s00134-011-2306-1. 21805160

[18] M. Fontaine , A. Pachot , A. Larue , et al., “Delayed Increase of S100A9 Messenger RNA Predicts Hospital‐Acquired Infection After Septic Shock,” Critical Care Medicine 39, no. 12 (2011): 2684–2690, 10.1097/CCM.0b013e3182282a40. 21765347

[19] C. Guignant , A. Lepape , X. Huang , et al., “Programmed Death‐1 Levels Correlate With Increased Mortality, Nosocomial Infection and Immune Dysfunctions in Septic Shock Patients,” Critical Care 15, no. 2 (2011): R99, 10.1186/cc10112. 21418617 PMC3219369

[20] K. Chang , C. Svabek , C. Vazquez‐Guillamet , et al., “Targeting the Programmed Cell Death 1: Programmed Cell Death Ligand 1 Pathway Reverses T Cell Exhaustion in Patients With Sepsis,” Critical Care 18, no. 1 (2014): R3, 10.1186/cc13176. 24387680 PMC4056005

[21] W. Y. Kwon , G. J. Suh , Y. S. Jung , et al., “Circulating Mitochondrial N‐Formyl Peptides Contribute to Secondary Nosocomial Infection in Patients With Septic Shock,” Proceedings of the National Academy of Sciences of the United States of America 118, no. 17 (2021): 11, 10.1073/pnas.2018538118. PMC8092466 33888581

[22] S. An , Y. Yao , H. B. Hu , et al., “PDHA1 Hyperacetylation‐Mediated Lactate Overproduction Promotes Sepsis‐Induced Acute Kidney Injury via Fis1 Lactylation,” Cell Death & Disease 14, no. 7 (2023): 13, 10.1038/s41419-023-05952-4. 37479690 PMC10362039

[23] S. Trzeciak , R. P. Dellinger , M. E. Chansky , et al., “Serum Lactate as a Predictor of Mortality in Patients With Infection,” Intensive Care Medicine 33, no. 6 (2007): 970–977, 10.1007/s00134-007-0563-9. 17431582

[24] M. Andersson , K. F. Schooner , V. K. Werther , et al., “Prehospital Lactate Analysis in Suspected Sepsis Improves Detection of Patients With Increased Mortality Risk: An Observational Study,” Critical Care 29, no. 1 (2025): 13, 10.1186/s13054-024-05225-2. 39838391 PMC11753079

[25] L. D. He , D. H. Yang , Q. Ding , Y. J. Su , and N. Ding , “Association Between Lactate and 28‐Day Mortality in Elderly Patients With Sepsis: Results From MIMIC‐IV Database,” Infectious Disease and Therapy 12, no. 2 (2023): 459–472, 10.1007/s40121-022-00736-3. PMC9925625 36520327

[26] J. E. Song , M. H. Kim , W. Y. Jeong , et al., “Mortality Risk Factors for Patients With Septic Shock After Implementation of the Surviving Sepsis Campaign Bundles,” Infection & Chemotherapy 48, no. 3 (2016): 199–208, 10.3947/ic.2016.48.3.199. 27659434 PMC5048001

[27] M. D. Howell , M. Donnino , P. Clardy , D. Talmor , and N. I. Shapiro , “Occult Hypoperfusion and Mortality in Patients With Suspected Infection,” Intensive Care Medicine 33, no. 11 (2007): 1892–1899, 10.1007/s00134-007-0680-5. 17618418

[28] B. Casserly , G. S. Phillips , C. Schorr , et al., “Lactate Measurements in Sepsis‐Induced Tissue Hypoperfusion: Results From the Surviving Sepsis Campaign Database,” Critical Care Medicine 43, no. 3 (2015): 567–573, 10.1097/ccm.0000000000000742. 25479113

[29] B. Yang , E. L. Norton , C. M. Rosati , et al., “Managing Patients With Acute Type A Aortic Dissection and Mesenteric Malperfusion Syndrome: A 20‐Year Experience,” Journal of Thoracic and Cardiovascular Surgery 158, no. 3 (2019): 675, 10.1016/j.jtcvs.2018.11.127. 30711274 PMC6570582

[30] T. C. Horan and T. G. Emori , “Definitions of Key Terms Used in the NNIS System,” American Journal of Infection Control 25, no. 2 (1997): 112–116, 10.1016/s0196-6553(97)90037-7. 9113287

[31] J. Leentjens , M. Kox , J. G. van der Hoeven , M. G. Netea , and P. Pickkers , “Immunotherapy for the Adjunctive Treatment of Sepsis: From Immunosuppression to Immunostimulation. Time for a Paradigm Change?,” American Journal of Respiratory and Critical Care Medicine 187, no. 12 (2013): 1287–1293, 10.1164/rccm.201301-0036CP. 23590272

[32] N. A. Hutchins , J. Unsinger , R. S. Hotchkiss , and A. Ayala , “The New Normal: Immunomodulatory Agents Against Sepsis Immune Suppression,” Trends in Molecular Medicine 20, no. 4 (2014): 224–233, 10.1016/j.molmed.2014.01.002. 24485901 PMC3976785

[33] R. S. Hotchkiss , G. Monneret , and D. Payen , “Immunosuppression in Sepsis: A Novel Understanding of the Disorder and a New Therapeutic Approach,” Lancet Infectious Diseases 13, no. 3 (2013): 260–268, 10.1016/s1473-3099(13)70001-x. 23427891 PMC3798159

[34] J. S. Boomer , K. To , K. C. Chang , et al., “Immunosuppression in Patients Who Die of Sepsis and Multiple Organ Failure,” JAMA 306, no. 23 (2011): 2594–2605, 10.1001/jama.2011.1829. 22187279 PMC3361243

[35] L. Nuyttens , M. Heyerick , G. Heremans , et al., “Unraveling Mitochondrial Pyruvate Dysfunction to Mitigate Hyperlactatemia and Lethality in Sepsis,” Cell Reports 44, no. 8 (2025): 26, 10.1016/j.celrep.2025.116032. 40694477

[36] O. R. Colegio , N. Q. Chu , A. L. Szabo , et al., “Functional Polarization of Tumour‐Associated Macrophages by Tumour‐Derived Lactic Acid,” Nature 513, no. 7519 (2014): 559, 10.1038/nature13490. 25043024 PMC4301845

[37] H. L. Ye , Q. B. Zhou , S. Y. Zheng , et al., “Tumor‐Associated Macrophages Promote Progression and the Warburg Effect via CCL18/NF‐kB/VCAM−1 Pathway in Pancreatic Ductal Adenocarcinoma,” Cell Death & Disease 9 (2018): 19, 10.1038/s41419-018-0486-0. 29670110 PMC5906621

[38] N. Liu , J. Luo , D. Kuang , et al., “Lactate Inhibits ATP6V0d2 Expression in Tumor‐Associated Macrophages to Promote HIF−2α‐Mediated Tumor Progression,” Journal of Clinical Investigation 129, no. 2 (2019): 631–646, 10.1172/jci123027. 30431439 PMC6355226

[39] J. Z. Wang , P. L. Yang , T. Y. Yu , et al., “Lactylation of PKM2 Suppresses Inflammatory Metabolic Adaptation in Pro‐Inflammatory Macrophages,” International Journal of Biological Sciences 18, no. 16 (2022): 6210–6225, 10.7150/ijbs.75434. 36439872 PMC9682528

[40] W. W. Shi , T. J. Cassmann , A. V. Bhagwate , et al., “Lactic Acid Induces Transcriptional Repression of Macrophage Inflammatory Response via Histone Acetylation,” Cell Reports 43, no. 2 (2024): 24, 10.1016/j.celrep.2024.113746. PMC10957222 38329873

[41] W. L. Ge , L. D. Meng , S. J. Cao , et al., “The SIX1/LDHA Axis Promotes Lactate Accumulation and Leads to NK Cell Dysfunction in Pancreatic Cancer,” Journal of Immunology Research 2023 (2023): 21, 10.1155/2023/6891636. PMC10022590 36937004

[42] A. Llibre , S. Kucuk , A. Gope , M. Certo , and C. Mauro , “Lactate: A Key Regulator of the Immune Response,” Immunity 58, no. 3 (2025): 535–554, 10.1016/j.immuni.2025.02.008. 40073846

[43] M. M. Fei , H. Zhang , F. B. Meng , et al., “Enhanced Lactate Accumulation Upregulates PD‐L1 Expression to Delay Neutrophil Apoptosis in Sepsis,” View‐China 5, no. 1 (2024): 18, 10.1002/viw.20230053.

[44] A. Angelin , L. Gil‐de‐Gómez , S. Dahiya , et al., “Foxp3 Reprograms T Cell Metabolism to Function in Low‐Glucose, High‐Lactate Environments,” Cell Metabolism 25, no. 6 (2017): 1282, 10.1016/j.cmet.2016.12.018. 28416194 PMC5462872

[45] M. D. Tyl , V. U. Merengwa , and I. M. Cristea , “Infection‐Induced Lysine Lactylation Enables Herpesvirus Immune Evasion,” Science Advances 11, no. 2 (2025): 20, 10.1126/sciadv.ads6215. PMC11708889 39772686

[46] C. J. Li , R. H. Zhou , G. Chen , X. C. Hao , and T. Zhu , “Knowledge Mapping and Research Hotspots of Artificial Intelligence on ICU and Anesthesia: From a Global Bibliometric Perspective,” Anesthesiology and Perioperative Science 1, no. 4 (2023): 13, 10.1007/s44254-023-00031-5.

