# Supplementary figures and images for: Association Between Lactate and ICU‐Acquired Infection in Critically Ill Patients With Sepsis: A Retrospective Study Using the MIMIC‐IV Database
Source: J Cell Mol Med. 2026 Mar 23;30(6):e71090. doi: 10.1111/jcmm.71090 (PMC13098033; doi:10.1111/jcmm.71090)

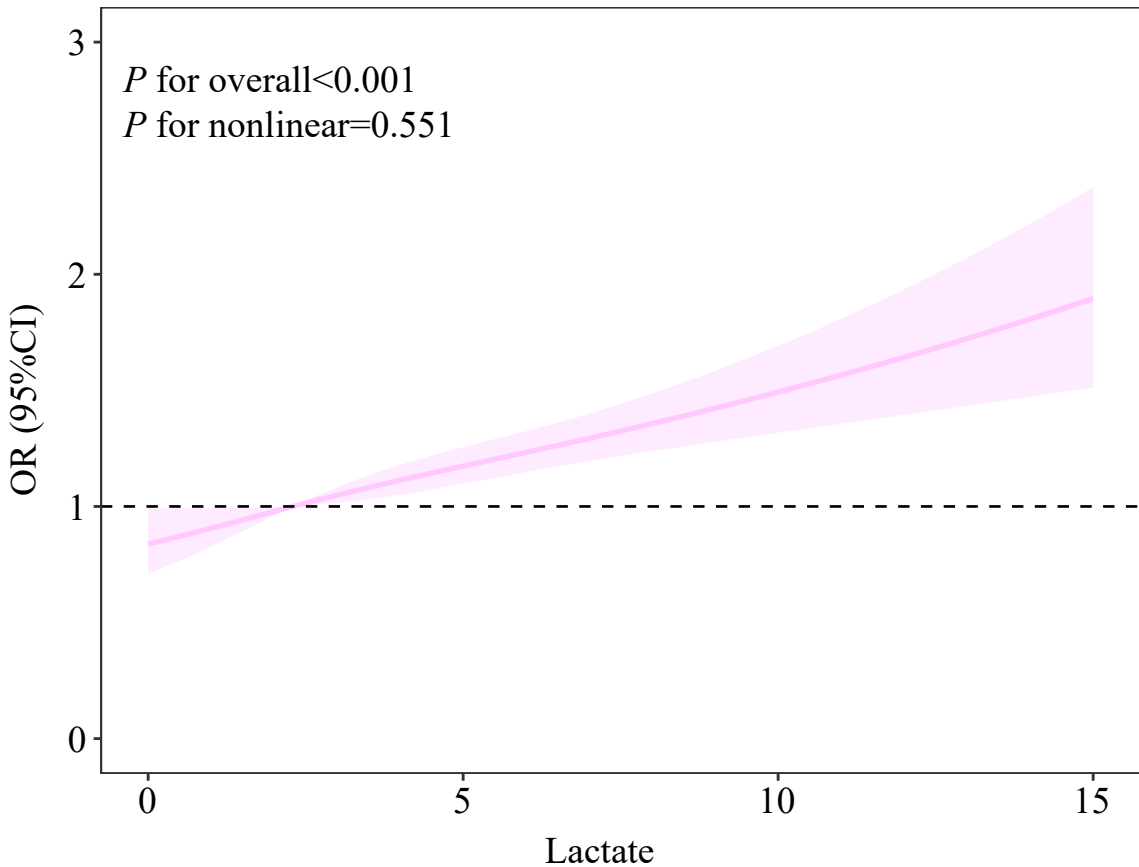

Supplement: Supplementary file 1 — Figure S1: Lactate and the estimated probability of first IAI occurrence (n = 1482). The restricted cubic spline curve illustrates the association between serum lactate levels (X‐axis, mmol/L) and the adjusted odds ratios (OR, with 95% CI) for the first IAI occurrence (Y‐axis). Shaded area represents 95% CI. OR = 1, lactate = 2.27 mmol/L. ICU, intensive care unit; CI, confidence interval; IAI, ICU‐acquired infection; OR, odds ratio. This analysis included 1482 patients with sepsis and IAI. [file JCMM-30-e71090-s002.pdf]

**A**

Total

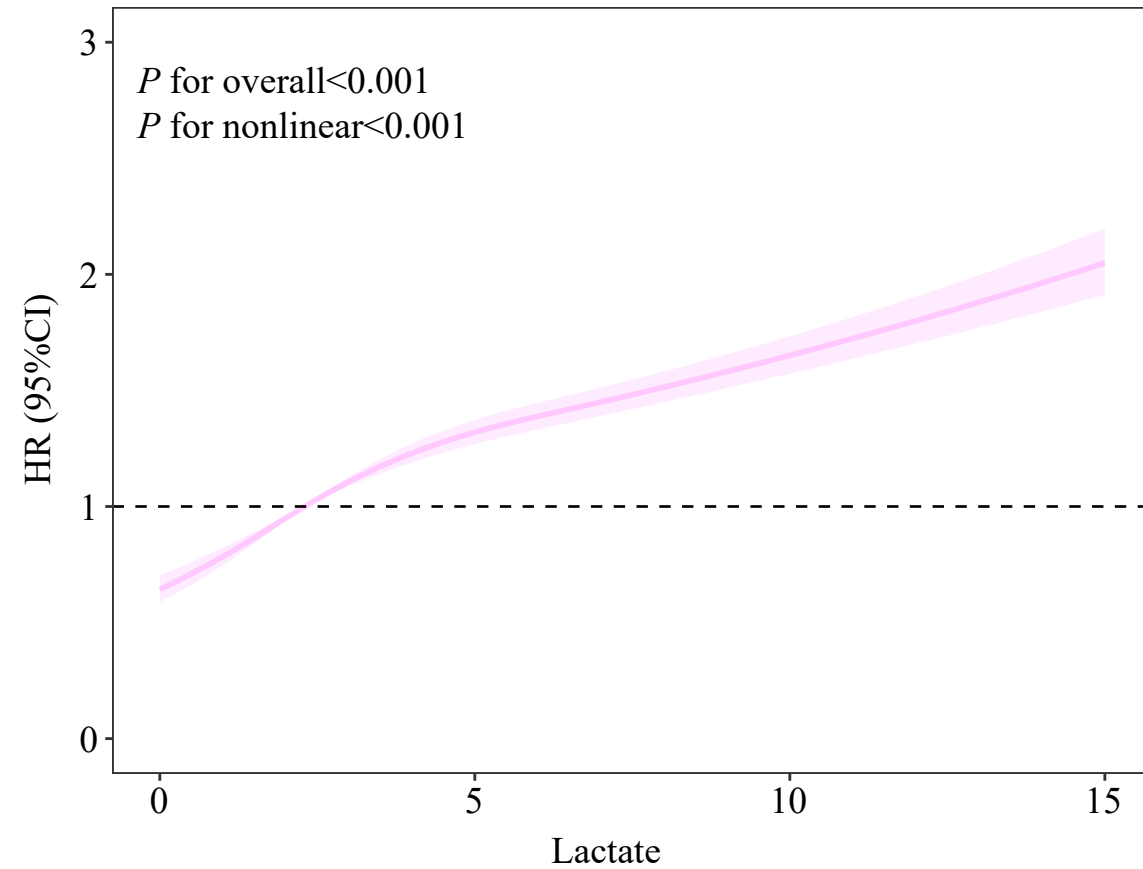**B**

Non-IAI

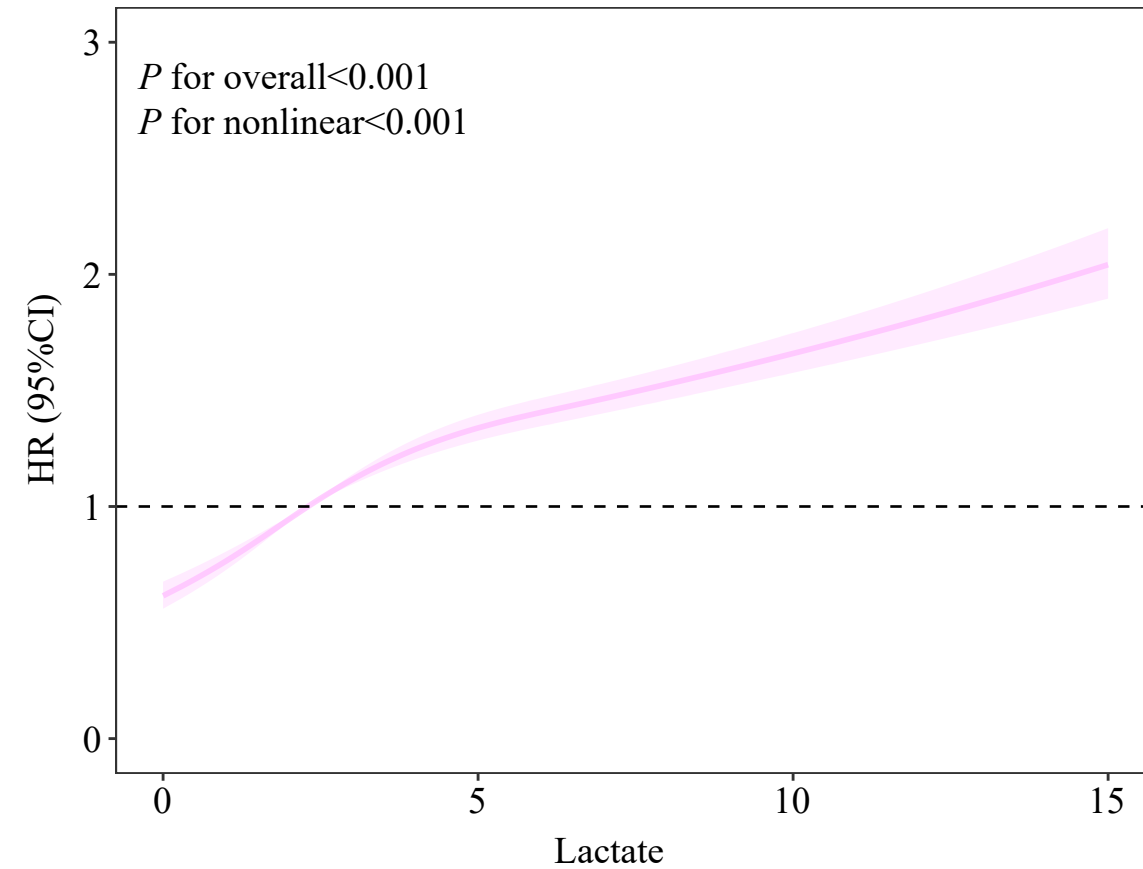**C**

IAI

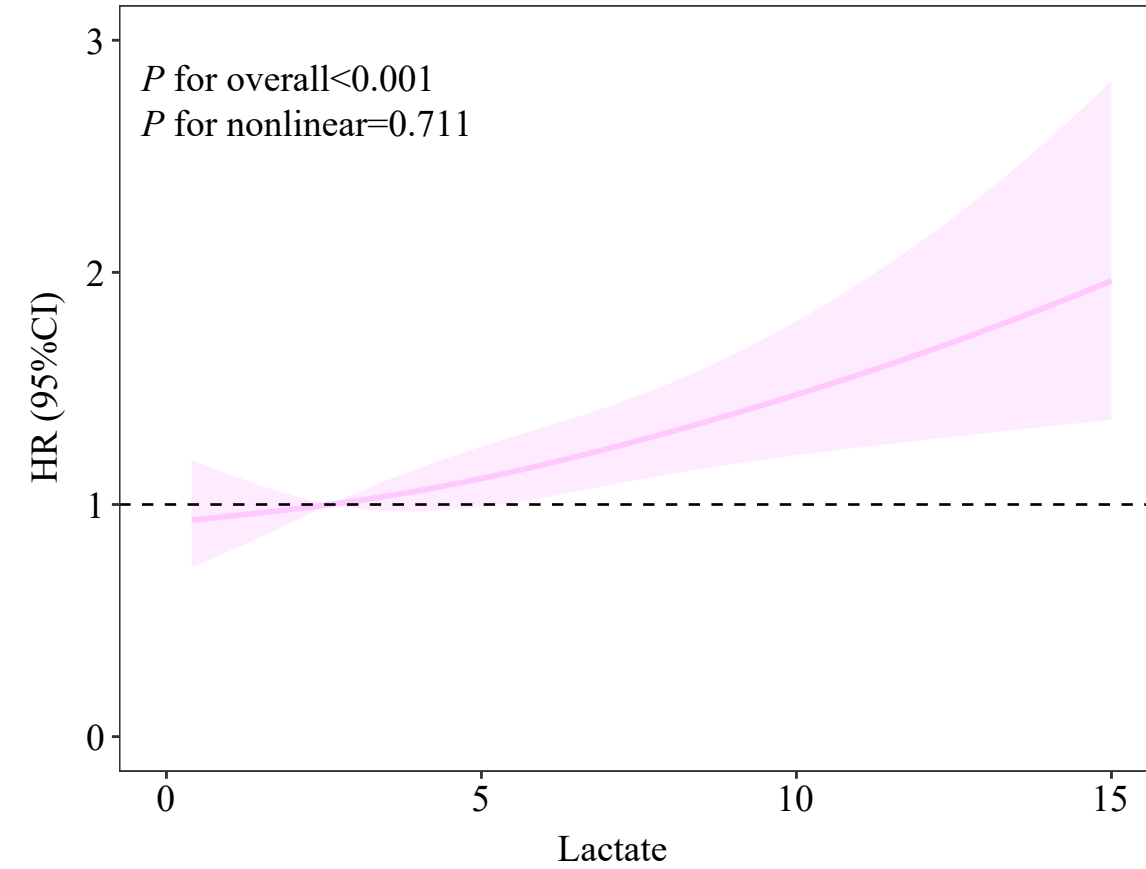

Supplement: Supplementary file 2 — Figure S2: Relationship between lactate and 28‐day mortality. The three restricted cubic spline curves display the association between serum lactate levels (X‐axis, mmol/L) and the adjusted hazard ratio (HR, with 95% CI) for 28‐day mortality (Y‐axis) across the specified patient populations. (A) In all sepsis populations, lactate was 2.27 mmol/L with HR = 1 (N = 17199). (B) In sepsis patients without IAI, lactate was 2.25 mmol/L with HR = 1 (n = 15179). (C) In sepsis patients with IAI, lactate was 2.62 mmol/L with HR = 1 (n = 1482). Shaded areas represent 95% CI. CI, confidence interval; HR, hazard ratio; IAI, ICU‐acquired infection; ICU, intensive care unit. This analysis included a total of 17199 patients with sepsis, of whom 1482 had an IAI. [file JCMM-30-e71090-s001.pdf]
