# Supplementary material for: Association Between Lactate and ICU‐Acquired Infection in Critically Ill Patients With Sepsis: A Retrospective Study Using the MIMIC‐IV Database
Source: J Cell Mol Med. 2026 Mar 23;30(6):e71090. doi: 10.1111/jcmm.71090 (PMC13098033; doi:10.1111/jcmm.71090)
Supplement: Supplementary file 3 — Table S1: Selection strategy for variables with multiple measurements. [file JCMM-30-e71090-s003.docx]

Table S1. Selection strategy for variables with multiple measurements

| Data items | Details |
| --- | --- |
| Lactate | Record the highest value for 24 hours of ICU admission |
| Temperature | Record the highest value for 24 hours of ICU admission |
| HR | Record the highest value for 24 hours of ICU admission |
| MBP | Record the lowest value for 24 hours of ICU admission |
| RR | Record the highest value for 24 hours of ICU admission |
| SpO2 | Record the lowest value for 24 hours of ICU admission |
| PH | Record the lowest value for 24 hours of ICU admission |
| PO2 | Record the lowest value for 24 hours of ICU admission |
| PCO2 | Record the highest value for 24 hours of ICU admission |
| BE | Record the lowest value for 24 hours of ICU admission |
| Anion gap | Record the highest value for 24 hours of ICU admission |
| Bicarbonate | Record the highest value for 24 hours of ICU admission |
| Hemoglobin | Record the lowest value for 24 hours of ICU admission |
| Platelet | Record the lowest value for 24 hours of ICU admission |
| WBC | Record the highest value for 24 hours of ICU admission |
| APTT | Record the highest value for 24 hours of ICU admission |
| PT | Record the highest value for 24 hours of ICU admission |
| INR | Record the highest value for 24 hours of ICU admission |
| Creatinine | Record the highest value for 24 hours of ICU admission |
| BUN | Record the highest value for 24 hours of ICU admission |
| Sodium | Record the highest value for 24 hours of ICU admission |
| Potassium | Record the highest value for 24 hours of ICU admission |
| Calcium | Record the lowest value for 24 hours of ICU admission |
| Chloride | Record the highest value for 24 hours of ICU admission |
| Glucose | Record the highest value for 24 hours of ICU admission |
| Abbreviations: ICU=intensive care unit; HR=heart rate; MBP=mean arterial pressure; RR=respiration rate; SpO2= percutaneous arterial oxygen saturation; PO2=partial pressure of oxygen; PCO2= partial pressure of carbon dioxide; BE=base excess; WBC= white blood cell; APTT= activated partial thromboplastin time; PT=prothrombin time; INR=international normalized ratio; BUN=blood urea nitrogen | |
