# Supplementary material for: Association Between Lactate and ICU‐Acquired Infection in Critically Ill Patients With Sepsis: A Retrospective Study Using the MIMIC‐IV Database
Source: J Cell Mol Med. 2026 Mar 23;30(6):e71090. doi: 10.1111/jcmm.71090 (PMC13098033; doi:10.1111/jcmm.71090)
Supplement: Supplementary file 4 — Table S2: Missing rate for variables extracted from MIMIC‐IV 3.0. [file JCMM-30-e71090-s005.docx]

Table S2. Missing rate for variables extracted from MIMIC-IV 3.0

| Variables | Missing, n/N^a^ | Missing (%) |
| --- | --- | --- |
| **Demographics** | | |
| Age | 0/17209 | 0.00 |
| Gender | 0/17209 | 0.00 |
| BMI | 4940/17209 | 28.71 |
| Ethnicity | 0/17209 | 0.00 |
| **Comorbidities** | | |
| Cardiovascular disease | 0/17209 | 0.00 |
| Neurological disease | 0/17209 | 0.00 |
| Pulmonary disease | 0/17209 | 0.00 |
| Liver disease | 0/17209 | 0.00 |
| Renal disease | 0/17209 | 0.00 |
| Diabetes | 0/17209 | 0.00 |
| Malignancy | 0/17209 | 0.00 |
| **Scores of severity** | | |
| CCI | 0/17209 | 0.00 |
| LODS | 0/17209 | 0.00 |
| OASIS | 0/17209 | 0.00 |
| APSIII | 0/17209 | 0.00 |
| SAPSII | 0/17209 | 0.00 |
| SIRS | 0/17209 | 0.00 |
| GCS | 2/17209 | 0.01 |
| SOFA | 0/17209 | 0.00 |
| **Vital signs** | | |
| Temperature | 945/17209 | 5.49 |
| HR | 0/17209 | 0.00 |
| MBP | 1/17209 | 0.01 |
| RR | 1/17209 | 0.01 |
| SpO2 | 1/17209 | 0.01 |
| **Laboratory results** | | |
| PH | 1/17209 | 0.01 |
| Lactate | 0/17209 | 0.00 |
| PO2 | 0/17209 | 0.00 |
| PCO2 | 0/17209 | 0.00 |
| BE | 0/17209 | 0.00 |
| Anion gap | 29/17209 | 0.17 |
| Bicarbonate | 7/17209 | 0.04 |
| Hemoglobin | 7/17209 | 0.04 |
| Platelet | 8/17209 | 0.05 |
| WBC | 9/17209 | 0.05 |
| APTT | 739/17209 | 4.29 |
| PT | 679/17209 | 3.95 |
| INR | 680/17209 | 3.95 |
| Creatinine | 4/17209 | 0.02 |
| BUN | 7/17209 | 0.04 |
| Sodium | 10/17209 | 0.06 |
| Potassium | 19/17209 | 0.11 |
| Calcium | 1080/17209 | 6.28 |
| Chloride | 7/17209 | 0.04 |
| Glucose | 58/17209 | 0.34 |
| **Treatment interventions** | | |
| Arterial catheter | 0/17209 | 0.00 |
| Central venous catheter | 0/17209 | 0.00 |
| Surgical drain | 0/17209 | 0.00 |
| Urinary catheter | 0/17209 | 0.00 |
| IMV | 0/17209 | 0.00 |
| RRT | 0/17209 | 0.00 |
| Glucocorticoid | 0/17209 | 0.00 |
| Immunosuppressant | 0/17209 | 0.00 |
| Antibiotic | 0/17209 | 0.00 |
| **Outcomes** | | |
| ICU-acquired infection | 0/17209 | 0.00 |
| LOS ICU | 0/17209 | 0.00 |
| LOS hospital | 0/17209 | 0.00 |
| In-hospital mortality | 0/17209 | 0.00 |
| ICU mortality | 0/17209 | 0.00 |
| 28-day mortality | 0/17209 | 0.00 |
| Abbreviations: BMI=body mass index; CCI=charlson comorbidity index; LODS=logistic organ dysfunction score; APSIII=acute physiology score III; SAPSII=simplified acute physiology score II;  SIRS=systemic inflammatory response syndrome score; GCS=glasgow coma scale; SOFA=sequential organ failure assessment; HR=heart rate; MBP=mean arterial pressure; RR=respiration rate; SpO2= percutaneous arterial oxygen saturation; PO2=partial pressure of oxygen; PCO2=partial pressure of carbon dioxide; BE=base excess; WBC=white blood cell; APTT=activated partial thromboplastin time; PT=prothrombin time; INR=international normalized ratio; BUN=blood urea nitrogen; IMV=invasive mechanical ventilation; RRT=renal replacement therapy; LOS=length of stay; ICU=intensive care unit | | |

^a^ Data for missing values are presented as n/N, where n is the number of missing cases and N is the total number of cases assessed.
