# Supplementary material for: Association Between Lactate and ICU‐Acquired Infection in Critically Ill Patients With Sepsis: A Retrospective Study Using the MIMIC‐IV Database
Source: J Cell Mol Med. 2026 Mar 23;30(6):e71090. doi: 10.1111/jcmm.71090 (PMC13098033; doi:10.1111/jcmm.71090)
Supplement: Supplementary file 5 — Table S3: Infection information of first IAI in patients admitted with sepsis classified according to lactate.a [file JCMM-30-e71090-s007.docx]

Table S3. Infection information of first IAI in patients admitted with sepsis classified according to lactate ^a^

| Variables | Total (0.4≤Lac≤32.0) | Q1 (Lac≤1.5) | Q2 (1.5<Lac≤2.0) | Q3 (2.0<Lac≤4.0) | Q4 (4.0<Lac≤6.0) | Q5 (Lac>6.0) | *P* value |
| --- | --- | --- | --- | --- | --- | --- | --- |
|  | (n=1482) | (n=369) | (n=207) | (n=436) | (n=199) | (n=271) |  |
| Days between ICU admission and first IAI, day | 5.3 (3.3-9.2) | 5.3 (3.1-9.6) | 5.2 (3.2-9.4) | 5.5 (3.5-9.3) | 5.1 (3.1-8.2) | 5.5 (3.4-9.1) | 0.670 |
| Sites of infection, n (%) |  |  |  |  |  |  |  |
| Respiratory | 871 (58.8) | 200 (54.2) | 114 (55.1) | 269 (61.7) | 132 (66.3) | 156 (57.6) | 0.030 |
| Bloodstream | 209 (14.1) | 41 (11.1) | 26 (12.6) | 58 (13.3) | 26 (13.1) | 58 (21.4) | 0.004 |
| Abdomen | 78 (5.3) | 21 (5.7) | 13 (6.3) | 25 (5.7) | 10 (5.0) | 9 (3.3) | 0.586 |
| Urinary | 154 (10.4) | 54 (14.6) | 26 (12.6) | 37 (8.5) | 17 (8.5) | 20 (7.4) | 0.010 |
| Skin | 46 (3.1) | 13 (3.5) | 10 (4.8) | 13 (3.0) | 3 (1.5) | 7 (2.6) | 0.377 |
| Neurological | 8 (0.5) | 3 (0.8) | 2 (1.0) | 3 (0.7) | 0 (0.0) | 0 (0.0) | 0.414 |
| Other/unknown | 126 (8.5) | 38 (10.3) | 17 (8.2) | 35 (8.0) | 13 (6.5) | 23 (8.5) | 0.614 |
| Isolated microorganisms, n (%) |  |  |  |  |  |  |  |
| Gram-positive bacteria | 711 (48.0) | 199 (53.9) | 99 (47.8) | 219 (50.2) | 89 (44.7) | 105 (38.7) | 0.003 |
| Gram-negative bacteria | 799 (53.9) | 183 (49.6) | 100 (48.3) | 233 (53.4) | 119 (59.8) | 164 (60.5) | 0.011 |
| Fungi | 97 (6.5) | 19 (5.1) | 14 (6.8) | 18 (4.1) | 16 (8.0) | 30 (11.1) | 0.004 |
| Virus | 14 (0.9) | 3 (0.8) | 3 (1.4) | 5 (1.1) | 0 (0.0) | 3 (1.1) | 0.593 |
| Unknown | 13 (0.9) | 7 (1.9) | 3 (1.4) | 2 (0.5) | 1 (0.5) | 0 (0.0) | 0.067 |
| Abbreviations: ICU=intensive care unit; IAI= ICU-acquired infection; Lac=lactate  ^a^ The unit of lactate is mmol/L | | | | | | | |
