# Supplementary material for: Association Between Lactate and ICU‐Acquired Infection in Critically Ill Patients With Sepsis: A Retrospective Study Using the MIMIC‐IV Database
Source: J Cell Mol Med. 2026 Mar 23;30(6):e71090. doi: 10.1111/jcmm.71090 (PMC13098033; doi:10.1111/jcmm.71090)
Supplement: Supplementary file 6 — Table S4: Detailed isolated microorganisms of first IAI in patients admitted with sepsis classified according to lactate.a [file JCMM-30-e71090-s004.docx]

Table S4. Detailed isolated microorganisms of first IAI in patients admitted with sepsis classified according to lactate ^a^

| Variables | Total (0.4≤Lac≤32.0) | Q1 (Lac≤1.5) | Q2 (1.5<Lac≤2.0) | Q3 (2.0<Lac≤4.0) | Q4 (4.0<Lac≤6.0) | Q5 (Lac>6.0) |
| --- | --- | --- | --- | --- | --- | --- |
|  | (n=1482) | (n=369) | (n=207) | (n=436) | (n=199) | (n=271) |
| **Gram-positive bacteria, n (%)** | **711 (48.0)** | **199 (53.9)** | **99 (47.8)** | **219 (50.2)** | **89 (44.7)** | **105 (38.7)** |
| Staphylococcus aureus | 359 (24.2) | 91 (24.7) | 54 (26.1) | 110 (25.2) | 58 (29.1) | 46 (17.0) |
| Coagulase-negative staphylococci | 106 (7.2) | 26 (7.0) | 16 (7.7) | 35 (8.0) | 11 (5.5) | 18 (6.6) |
| Streptococcus pneumoniae | 14 (0.9) | 5 (1.4) | 3 (1.4) | 3 (0.7) | 1 (0.5) | 2 (0.7) |
| Other streptococci | 29 (0.2) | 11 (3.0) | 0 (0.0) | 10 (2.3) | 3 (1.5) | 5 (1.8) |
| Enterococcus | 95 (6.4) | 33 (8.9) | 11 (5.3) | 28 (6.4) | 8 (4.0) | 15 (5.5) |
| Other/unknown | 138 (9.3) | 43 (11.7) | 20 (9.7) | 41 (9.4) | 11 (5.5) | 23 (8.5) |
| **Gram-negative bacteria, n (%)** | **799 (53.9)** | **183 (49.6)** | **100 (48.3)** | **233 (53.4)** | **119 (59.8)** | **164 (60.5)** |
| Escherichia coli | 129 (8.7) | 31 (8.4) | 16 (7.7) | 37 (8.5) | 19 (9.5) | 26 (9.6) |
| Enterobacter | 106 (7.2) | 17 (4.6) | 16 (7.7) | 36 (8.3) | 15 (7.5) | 22 (8.1) |
| Klebsiella | 129 (8.7) | 25 (6.8) | 16 (7.7) | 35 (8.0) | 20 (10.1) | 33 (12.2) |
| Pseudomonas | 181 (12.2) | 46 (12.5) | 26 (12.6) | 52 (11.9) | 24 (12.1) | 33 (12.2) |
| Acinetobacter | 33 (2.2) | 9 (2.4) | 2 (1.0) | 10 (2.3) | 7 (3.5) | 5 (1.8) |
| Proteus | 25 (1.7) | 6 (1.6) | 5 (2.4) | 6 (1.4) | 4 (2.0) | 4 (1.5) |
| Stenotrophomonas | 70 (4.7) | 15 (4.1) | 14 (6.8) | 19 (4.4) | 8 (4.0) | 14 (5.2) |
| Serratia | 57 (3.8) | 10 (2.7) | 8 (3.9) | 16 (3.7) | 9 (4.5) | 14 (5.2) |
| Hemophilus | 39 (2.6) | 11 (3.0) | 3 (1.4) | 12 (2.8) | 5 (2.5) | 8 (3.0) |
| Citrobacter | 25 (1.7) | 8 (2.2) | 4 (1.9) | 4 (0.9) | 7 (3.5) | 2 (0.7) |
| Morganella | 5 (0.3) | 1 (0.3) | 0 (0.0) | 2 (0.5) | 1 (0.5) | 1 (0.4) |
| Hafnia | 3 (0.2) | 0 (0.0) | 0 (0.0) | 0 (0.0) | 0 (0.0) | 3 (1.1) |
| Other/unknown | 125 (8.4) | 29 (7.9) | 12 (5.8) | 36 (8.3) | 22 (11.1) | 26 (9.6) |
| **Fungi, n (%)** | **97 (6.5)** | **19 (5.1)** | **14 (6.8)** | **18 (4.1)** | **16 (8.0)** | **30 (11.1)** |
| Candida albicans | 31 (2.1) | 7 (1.9) | 3 (1.4) | 6 (1.4) | 7 (3.5) | 8 (3.0) |
| Candida non albicans | 36 (2.4) | 8 (2.2) | 4 (1.9) | 4 (0.9) | 7 (3.5) | 13 (4.8) |
| Aspergillus | 28 (1.9) | 5 (1.4) | 7 (3.4) | 6 (1.4) | 3 (1.5) | 7 (2.6) |
| Other | 6 (0.4) | 1 (0.3) | 0 (0.0) | 3 (0.7) | 0 (0.0) | 2 (0.7) |
| **Virus, n (%)** | **14 (0.9)** | **3 (0.8)** | **3 (1.4)** | **5 (1.1)** | **0 (0.0)** | **3 (1.1)** |
| **Unknown, n (%)** | **13 (0.9)** | **7 (1.9)** | **3 (1.4)** | **2 (0.5)** | **1 (0.5)** | **0 (0.0)** |
| Abbreviations: ICU=intensive care unit; IAI= ICU-acquired infection; Lac=lactate  ^a^ The unit of lactate is mmol/L | | | | | | |
