# Supplementary material for: Association Between Lactate and ICU‐Acquired Infection in Critically Ill Patients With Sepsis: A Retrospective Study Using the MIMIC‐IV Database
Source: J Cell Mol Med. 2026 Mar 23;30(6):e71090. doi: 10.1111/jcmm.71090 (PMC13098033; doi:10.1111/jcmm.71090)
Supplement: Supplementary file 7 — Table S5: Detailed infection information of all IAI in patients admitted with sepsis classified according to lactate.a [file JCMM-30-e71090-s008.docx]

Table S5. Detailed infection information of all IAI in patients admitted with sepsis classified according to lactate ^a^

|  | Total (0.4≤Lac≤32.0) | Q1 (Lac≤1.5) | Q2 (1.5<Lac≤2.0) | Q3 (2.0<Lac≤4.0) | Q4 (4.0<Lac≤6.0) | Q5 (Lac>6.0) |
| --- | --- | --- | --- | --- | --- | --- |
| Variables | (n=1482) | (n=369) | (n=207) | (n=436) | (n=199) | (n=271) |
| **Sites of infection, n (%)** |  |  |  |  |  |  |
| Respiratory | 922 (62.2) | 211 (57.2) | 121 (58.5) | 284 (65.1) | 139 (69.8) | 167 (61.6) |
| Bloodstream | 257 (17.3) | 53 (14.4) | 33 (15.9) | 72 (16.5) | 31 (15.6) | 68 (25.1) |
| Abdomen | 100 (6.7) | 29 (7.9) | 16 (7.7) | 28 (6.4) | 13 (6.5) | 14 (5.2) |
| Urinary | 188 (12.7) | 63 (17.1) | 34 (16.4) | 43 (9.9) | 21 (10.6) | 27 (10.0) |
| Skin | 67 (4.5) | 18 (4.9) | 12 (5.8) | 17 (3.9) | 8 (4.0) | 12 (4.4) |
| Neurological | 10 (0.7) | 3 (0.8) | 2 (1.0) | 5 (1.1) | 0 (0.0) | 0 (0.0) |
| Other/unknown | 162 (10.9) | 45 (12.2) | 21 (10.1) | 5 (1.1) | 18 (9.0) | 31 (11.4) |
| **Isolated microorganisms, n (%)** |  |  |  |  |  |  |
| Gram-positive bacteria | 780 (52.6) | 217 (58.8) | 110 (53.1) | 235 (53.9) | 100 (50.3) | 118 (43.5) |
| Staphylococcus aureus | 389 (26.2) | 104 (28.2) | 59 (28.5) | 115 (26.4) | 60 (30.2) | 51 (18.8) |
| Coagulase-negative staphylococci | 137 (9.2) | 35 (9.5) | 22 (10.6) | 44 (10.1) | 13 (6.5) | 23 (8.5) |
| Streptococcus pneumoniae | 16 (1.1) | 7 (1.9) | 3 (1.4) | 3 (0.7) | 1 (0.5) | 2 (0.7) |
| Other streptococci | 31 (2.1) | 11 (3.0) | 0 (0.0) | 12 (2.8) | 3 (1.5) | 5 (1.8) |
| Enterococcus | 114 (7.7) | 36 (9.8) | 13 (6.3) | 33 (7.6) | 14 (7.0) | 18 (6.6) |
| Other/unknown | 155 (10.5) | 49 (13.3) | 21 (10.1) | 43 (9.9) | 16 (8.0) | 26 (9.6) |
| Gram-negative bacteria | 855 (57.7) | 196 (53.1) | 109 (52.7) | 256 (58.7) | 123 (61.8) | 171 (63.1) |
| Escherichia coli | 144 (9.7) | 32 (8.7) | 17 (8.2) | 46 (10.6) | 21 (10.6) | 28 (10.3) |
| Enterobacter | 127 (8.6) | 22 (6.0) | 20 (9.7) | 43 (9.9) | 16 (8.0) | 26 (9.6) |
| Klebsiella | 146 (9.9) | 32 (8.7) | 17 (8.2) | 42 (9.6) | 21 (10.6) | 34 (12.5) |
| Pseudomonas | 203 (13.7) | 49 (13.3) | 33 (15.9) | 60 (13.8) | 25 (12.6) | 36 (13.3) |
| Acinetobacter | 35 (2.4) | 10 (2.7) | 2 (1.0) | 11 (2.5) | 7 (3.5) | 5 (1.8) |
| Proteus | 31 (2.1) | 7 (1.9) | 7 (3.4) | 8 (1.8) | 5 (2.5) | 4 (1.5) |
| Stenotrophomonas | 83 (5.6) | 19 (5.1) | 14 (6.8) | 23 (5.3) | 10 (5.0) | 17 (6.3) |
| Serratia | 64 (4.3) | 13 (3.5) | 10 (4.8) | 16 (3.7) | 10 (5.0) | 15 (5.5) |
| Hemophilus | 47 (3.2) | 11 (3.0) | 4 (1.9) | 14 (3.2) | 7 (3.5) | 11 (4.1) |
| Citrobacter | 29 (2.0) | 8 (2.2) | 4 (1.9) | 5 (1.1) | 7 (3.5) | 5 (1.8) |
| Morganella | 5 (0.3) | 1 (0.3) | 0 (0.0) | 2 (0.5) | 1 (0.5) | 1 (0.4) |
| Hafnia | 3 (0.2) | 0 (0.0) | 0 (0.0) | 0 (0.0) | 0 (0.0) | 3 (1.1) |
| Other/unknown | 130 (8.8) | 28 (7.6) | 13 (6.3) | 37 (8.5) | 23 (11.6) | 29 (10.7) |
| Fungi | 127 (8.6) | 22 (6.0) | 18 (8.7) | 24 (5.5) | 23 (11.6) | 40 (14.8) |
| Candida albicans | 44 (3.0) | 9 (2.4) | 5 (2.4) | 10 (2.3) | 8 (4.0) | 12 (4.4) |
| Candida non albicans | 48 (3.2) | 9 (2.4) | 7 (3.4) | 5 (1.1) | 11 (5.5) | 16 (5.9) |
| Aspergillus | 34 (2.3) | 5 (1.4) | 7 (3.4) | 7 (1.6) | 4 (2.0) | 11 (4.1) |
| Other | 7 (0.5) | 1 (0.3) | 0 (0.0) | 3 (0.7) | 1 (0.5) | 2 (0.7) |
| Virus | 17 (1.1) | 3 (0.8) | 4 (1.9) | 5 (1.1) | 1 (0.5) | 4 (1.5) |
| Unknown | 10 (0.7) | 6 (1.6) | 2 (1.0) | 1 (0.2) | 1 (0.5) | 0 (0.0) |
| Abbreviations: ICU=intensive care unit; IAI= ICU-acquired infection; Lac=lactate  ^a^ The unit of lactate is mmol/L | | | | | | |
