# Supplementary material for: Association Between Lactate and ICU‐Acquired Infection in Critically Ill Patients With Sepsis: A Retrospective Study Using the MIMIC‐IV Database
Source: J Cell Mol Med. 2026 Mar 23;30(6):e71090. doi: 10.1111/jcmm.71090 (PMC13098033; doi:10.1111/jcmm.71090)
Supplement: Supplementary file 8 — Table S6: ICU and in‐hospital mortality in different lactate categories with or without IAI. [file JCMM-30-e71090-s006.docx]

Table S6. ICU and in-hospital mortality in different lactate categories with or without IAI

|  |  | Unadjusted model | |  | Model 1 ^a^ | |  | Model 2 ^b^ | |  | Model 3 ^c^ | |
| --- | --- | --- | --- | --- | --- | --- | --- | --- | --- | --- | --- | --- |
|  |  | OR (95%CI) | *P* value |  | OR (95%CI) | *P* value |  | OR (95%CI) | *P* value |  | OR (95%CI) | *P* value |
| ICU mortality |  |  |  |  |  |  |  |  |  |  |  |  |
| Without IAI |  |  |  |  |  |  |  |  |  |  |  |  |
| Q1 | 403/4133 (9.8) | Reference |  |  | Reference |  |  | Reference |  |  | Reference |  |
| Q2 | 311/2564 (12.1) | 1.28 (1.09-1.49) | 0.002 |  | 1.27 (1.08-1.49) | 0.003 |  | 1.17 (0.99-1.38) | 0.059 |  | 1.17 (0.99-1.38) | 0.057 |
| Q3 | 736/5478 (13.4) | 1.44 (1.26-1.64) | <0.001 |  | 1.43 (1.26-1.63) | <0.001 |  | 1.24 (1.07-1.42) | 0.003 |  | 1.23 (1.07-1.42) | 0.004 |
| Q4 | 302/1805 (16.7) | 1.86 (1.58-2.18) | <0.001 |  | 1.89 (1.61-2.22) | <0.001 |  | 1.29 (1.07-1.55) | 0.007 |  | 1.29 (1.07-1.56) | 0.006 |
| Q5 | 516/1747 (29.5) | 3.88 (3.36-4.49) | <0.001 |  | 4.04 (3.49-4.67) | <0.001 |  | 1.88 (1.53-2.30) | <0.001 |  | 1.87 (1.52-2.29) | <0.001 |
| With IAI |  |  |  |  |  |  |  |  |  |  |  |  |
| Q1 | 74/369 (20.1) | Reference |  |  | Reference |  |  | Reference |  |  | Reference |  |
| Q2 | 42/208 (20.3) | 1.01 (0.66-1.54) | 0.946 |  | 1.03 (0.67-1.57) | 0.897 |  | 0.96 (0.61-1.49) | 0.845 |  | 0.96 (0.61-1.50) | 0.862 |
| Q3 | 99/436 (22.7) | 1.17 (0.84-1.65) | 0.362 |  | 1.19 (0.84-1.67) | 0.330 |  | 0.95 (0.65-1.39) | 0.802 |  | 0.95 (0.65-1.39) | 0.804 |
| Q4 | 44/199 (22.1) | 1.13 (0.74-1.72) | 0.565 |  | 1.21 (0.79-1.85) | 0.373 |  | 0.79 (0.48-1.30) | 0.360 |  | 0.80 (0.48-1.32) | 0.383 |
| Q5 | 87/271 (32.1) | 1.88 (1.32-2.71) | 0.001 |  | 1.92 (1.33-2.77) | <0.001 |  | 0.83 (0.48-1.42) | 0.502 |  | 0.84 (0.49-1.44) | 0.525 |
| In-hospital mortality |  |  |  |  |  |  |  |  |  |  |  |  |
| Without IAI |  |  |  |  |  |  |  |  |  |  |  |  |
| Q1 | 628/4133 (15.2) | Reference |  |  | Reference |  |  | Reference |  |  | Reference |  |
| Q2 | 487/2564 (19.0) | 1.31 (1.15-1.49) | <0.001 |  | 1.30 (1.14-1.48) | <0.001 |  | 1.20 (1.05-1.38) | 0.009 |  | 1.22 (1.06-1.40) | 0.006 |
| Q3 | 1042/5478 (19.0) | 1.31 (1.18-1.46) | <0.001 |  | 1.31 (1.17-1.46) | <0.001 |  | 1.13 (1.01-1.28) | 0.040 |  | 1.16 (1.03-1.31) | 0.018 |
| Q4 | 417/1805 (23.1) | 1.68 (1.46-1.93) | <0.001 |  | 1.72 (1.50-1.98) | <0.001 |  | 1.20 (1.02-1.41) | 0.030 |  | 1.24 (1.05-1.45) | 0.011 |
| Q5 | 631/1747 (36.1) | 3.16 (2.77-3.59) | <0.001 |  | 3.36 (2.95-3.83) | <0.001 |  | 1.66 (1.38-2.00) | <0.001 |  | 1.70 (1.41-2.04) | <0.001 |
| With IAI |  |  |  |  |  |  |  |  |  |  |  |  |
| Q1 | 90/369 (24.4) | Reference |  |  | Reference |  |  | Reference |  |  | Reference |  |
| Q2 | 53/208 (25.6) | 1.07 (0.72-1.58) | 0.746 |  | 1.08 (0.73-1.61) | 0.690 |  | 1.02 (0.67-1.55) | 0.917 |  | 1.03 (0.67-1.56) | 0.899 |
| Q3 | 121/436 (27.8) | 1.19 (0.87-1.64) | 0.280 |  | 1.21 (0.88-1.67) | 0.244 |  | 1.03 (0.72-1.47) | 0.873 |  | 1.03 (0.72-1.48) | 0.856 |
| Q4 | 56/199 (28.1) | 1.21 (0.82-1.79) | 0.330 |  | 1.32 (0.88-1.95) | 0.174 |  | 0.98 (0.61-1.56) | 0.923 |  | 0.98 (0.61-1.56) | 0.921 |
| Q5 | 96/271 (35.4) | 1.70 (1.21-2.40) | 0.002 |  | 1.73 (1.22-2.46) | 0.002 |  | 0.87 (0.52-1.45) | 0.584 |  | 0.87 (0.52-1.46) | 0.601 |
| Abbreviations: ICU=intensive care unit; IAI=ICU-acquired infection; OR=odds ratio; CI=confidence interval  ^a^ Adjusted for age and gender  ^b^ Adjusted for comorbidities (cardiovascular disease, pulmonary disease, liver disease, renal disease and malignancy), scores of severity (CCI, LODS, OASIS, APSIII,  SAPSII and SOFA), vital signs (temperature and SpO2), and laboratory results (PH, PO2, BE, bicarbonate, anion gap, hemoglobin, platelet, APTT, PT, INR, creatinine, BUN,  potassium, glucose and chloride) based on model 1  ^c^ Adjusted for treatment interventions (arterial catheter, urinary catheter, IMV and RRT) based on model 2 | | | | | | | | | | | | |
